# Identification of hub genes in congenital hypothyroidism and construction of the associated immune regulatory network

**DOI:** 10.3389/fimmu.2025.1608098

**Published:** 2025-10-15

**Authors:** Mingliang Shan, Li Xu, Wenzhe Yang, Shiguo Liu

**Affiliations:** ^1^ Medical Genetic Department, The Affiliated Hospital of Qingdao University, Qingdao, China; ^2^ Gaomi Maternity and Child Health Hospital, Gaomi, China; ^3^ Shandong Second Medical University, Weifang, China; ^4^ Shandong University of Traditional Chinese Medicine, Jinan, China

**Keywords:** CH, hub gene, bioinformatics, machine learning, Mendelian randomization (MR), IL-2

## Abstract

**Background:**

The absence of universal diagnosis and treatment recommendations for congenital hypothyroidism (CH) has led to suboptimal diagnostic and therapeutic outcomes. This study aimed to provide immunologically relevant evidence for the diagnosis and treatment of CH.

**Methods:**

Datasets related to CH were selected. Differentially expressed genes were screened, followed by enrichment analysis, weighted gene coexpression network analysis (WGCNA), protein–protein interaction analysis, and machine learning for the identification of hub genes. The reliability of these hub genes was verified through least absolute shrinkage and selection operator (LASSO) regression, box plot comparison, and receiver operating characteristic (ROC) curve analysis. Through gene set enrichment analysis (GSEA) of coexpressed genes, the common pathways of the hub genes and the inflammatory factors involved were identified. Immunoinfiltration analysis was carried out to verify the immunological correlation. Inflammatory factors and immune cells were screened by batch Mendelian randomization. Finally, the reliability of the hub genes, their relationships with inflammatory factors, and their impacts on cell function and the synthesis of free thyroxine (FT4) were validated through real-time quantitative polymerase chain reaction (RT–qPCR), enzyme-linked immunosorbent assay (ELISA), and cell proliferation experiments.

**Results:**

Multiomics analysis confirmed that APP, DDB1, MRPS5, and MRPL33 were hub genes with low expression levels in CH. These genes negatively regulated IL-2, and subsequently, through the STAT5 and MTORC1 pathways, they positively regulated (CD27 on IgD- CD38+ B cells) and (CD27 on switched memory B cells)/CD244 and negatively regulated (CD33dim HLA DR+ CD11b- Absolute Count)/IL-18 and (CD28+ CD4-CD8- T-cell %T cell)/OPG, promoting the progression of CH. Increasing the expression levels of these hub genes could increase the activity of thyroid cells and promote the synthesis of FT4 through the abovementioned pathways.

**Conclusions:**

APP, DDB1, MRPS5, and MRPL33 regulate the expression of IL-2, act on relevant immune cell subtypes through the STAT5 and MTORC1 pathways, negatively regulate IL-18 and OPG, and positively regulate CD244, thereby influencing the activity of thyroid cells and the synthesis of FT4.

## 1 Introduction

CH is a common endocrine and metabolic disorder that typically manifests in the neonatal period. It is characterized by a marked deficiency in thyroid hormone secretion (1, 2), with an incidence rate ranging from 1 per 3,000 to 1 per 4,000 (3, 4). When the level of thyroid hormone secretion falls below the normal range, children may experience metabolic disorders, weakened physiological functions, growth retardation, and intellectual disability (5). The diagnosis of hypothyroidism is based on a decrease in the serum thyroid hormone concentration and a significant increase in thyroid-stimulating hormone concentration (6). Hypothyroidism, which occurs in the first few months after birth, can cause irreversible damage to the central nervous system. Therefore, CH must be diagnosed and treated promptly, as it is a common and preventable cause of intellectual disability (7, 8). Identifying hub genes associated with CH and elucidating their mechanisms are crucial for the early diagnosis and treatment of CH (9, 10).

CH is classified into thyroid dysgenesis and hormone secretion disorders (11, 12). Thyroid dysgenesis is mainly categorized into ectopic thyroid and congenital thyroid hemiagenesis (13). Evidence suggests that NKX2-1 (14) and FOXE1 (15) are key transcription factors contributing to thyroid dysgenesis. Hormonal secretion disorders are mostly caused by the underdevelopment of the hypothalamic-pituitary-thyroid (HPT) axis, which is particularly prominent in preterm infants (16).It is caused mainly by defects in the molecular pathways of thyroid hormone production, resulting in a structurally intact gland being unable to produce hormones (17, 18). However, the specific mechanisms underlying the impact on molecular pathways have not been fully elucidated (19). Therefore, further research on the mechanisms of CH is highly important.

Hypothyroidism, which shares similar clinical manifestations with CH, is an autoimmune disease that often co-occurs with other autoimmune diseases, such as type 1 diabetes, atrophic gastritis, celiac disease, and multiple autoimmune endocrinopathies (20). Autoimmune thyroid diseases are caused by immune system disorders, in which the immune system initiates an immune attack against the thyroid gland. These diseases are mainly T-cell-mediated Graves’ disease and Hashimoto’s thyroiditis, and both, like CH, are characterized mainly by hypothyroidism (21, 22). These findings strongly suggest that there is a close association between immunity and hypothyroidism (23). Therefore, determining whether there is an immune-related pathogenesis in CH is the focus of this study.

In this study, we aimed to identify hub genes and determine the immune regulatory network related to the pathogenesis of CH. First, bioinformatics, machine learning, and batch Mendelian randomization (MR) were used to screen and validate the hub genes and the related immune regulatory network. *In vitro* experiments were subsequently conducted to verify the expression of the hub genes, their connection with immune regulation, and their impacts on cell function and FT4 synthesis. These findings provide evidence to further clarify the association between CH and immune disorders.

## 2 Materials and methods

### 2.1 Bioinformatics analysis

#### 2.1.1 Data sources

The GSE18152 dataset was selected from the GEO database (https://www.ncbi.nlm.nih.gov/geo/) as CH samples, which includes 80 thyroid samples from CH patients (57 females and 23 males, all neonatal samples) analyzed using array comparative genomic hybridization (CGH) technology. The GTEx dataset (comprising 11 datasets), consisting of 366 normal thyroid samples (135 females and 231 males with unknown age groups) analyzed by RNA sequencing, was selected from the UCSC Xena website (http://xena.ucsc.edu/) as normal samples (279 normal thyroid samples). After merging the two datasets (named MERGE), batch correction was performed using the normalizeBetweenArrays function from the limma package.

#### 2.1.2 Screening of differentially expressed genes and functional enrichment analysis

The limma package in R software (version 4.4.1) was used to screen differentially expressed genes (DEGs). The selection criteria were P < 0.05 and |log2 FC| > 2. After log transformation, this indicates that the difference in expression between the two groups is more than 4-fold. Since the preliminary analysis with |log2 FC| > 1 yielded a large number of genes, we therefore chose more stringent criteria. GO, KEGG, and immune-related GSEA enrichment analyses were carried out on the DEGs. The selection criterion was P < 0.05, and genes with |log2 FC| > 1 were additionally selected for GSEA.

#### 2.1.3 Weighted gene coexpression network analysis

First, cluster analysis was performed on the samples, and then the outlier data were removed. The fitting index is positively correlated with the power value, while the average connectivity is negatively correlated with the power value. To obtain the maximum fitting index and average connectivity, the optimal power value calculated automatically by the function is 11. The fitting index and average connectivity were determined to construct the optimal scale-free network, and a topological relationship diagram was drawn to confirm the successful construction of the network. Genes were clustered on the basis of the obtained distance matrix. The modules were subsequently clustered according to the condition that the number of genes in the dynamic module was ≥ 30, and modules with high correlation were subsequently clustered and merged. Important modules were screened by drawing heatmaps of the relationships between modules and clinical features and generating gene importance plots. Genes with gene significance (GS) > 0.5 and module membership (MM) > 0.8 were selected as the key module characteristic gene set.

#### 2.1.4 Identification of hub genes

1. The JSVM-RFE algorithm in machine learning was used to screen characteristic genes.
2. The DEGs were uploaded to STRING (https://string-db.org/). Protein–protein interaction (PPI) and mapping diagrams were obtained with a confidence level of 0.4, and genes with more than 10 connections were selected as characteristic genes.
3. The MCC algorithm in the cytoHubba plugin of Cytoscape was utilized to screen the 20 characteristic genes with the highest connectivity.

Finally, the above three sets of characteristic genes were intersected with the characteristic gene set obtained from WGCNA to obtain the hub genes.

#### 2.1.5 Validation of hub genes

1. Validation was carried out using LASSO regression analysis.
2. Through box plots of the CH group and the normal group, the differences in the expression of the hub genes between the two groups were compared.
3. The receiver operating characteristic (ROC) curve was plotted, and an area under the curve (AUC) > 0.8 was selected as the validation criterion.

#### 2.1.6 GSEA of coexpressed genes

Through GSEA of coexpressed genes, gene sets that had synergistic effects on each hub gene were selected from the MERGE dataset. The enrichment pathways of each hub gene were screened on the basis of these gene sets. The intersections of these pathways were used to identify the commonly enriched inflammatory factors and pathways of the hub genes.

#### 2.1.7 Immunoinfiltration analysis

Immune-related single-sample gene set enrichment analysis (ssGSEA) and CIBERSORT immune cell infiltration analysis were conducted on the hub genes. These two complementary analyses can be combined to investigate whether there are immune differences in CH and whether there is a close association between the hub genes and immune cells.

### 2.2 Batch MR analysis

In accordance with the TROBE-MR checklist-fillable (24, 25), we used the TwosampleMR package in R (version 4.4.1) to conduct MR analysis to identify immune cells and inflammatory factors associated with CH. All the data were derived from publicly available databases, and all the original studies obtained ethical approval.

#### 2.2.1 Data sources

Data on CH were obtained from the IEU database (https://gwas.mrcieu.ac.uk/), specifically for hypothyroidism (congenital or acquired) (GWAS ID: finn-b-HYPOTHYROIDISM, containing 16,378,441 single nucleotide polymorphisms (SNPs)). A total of 728 types of immune cells were sourced from the EBI GWAS Catalog database (https://www.ebi.ac.uk/gwas/), corresponding to the GWAS ID (**Supplementary Tables S1**-728). Ninety types of inflammatory factor data were obtained from the EBI GWAS Catalog, with corresponding IDs (**Supplementary Tables S1**-90). Data on IL-2 were obtained from the IEU database (GWAS ID: prot-c-3070_1_2, containing 501,428 SNPs).

#### 2.2.2 Instrumental variable selection and data harmonization

First, genome-wide significant SNPs (P < 5×10-8) were included. If there were no significant SNPs available as instrumental variables (IVs), SNPs with a P value less than the genome-wide significance level (P < 5×10-6) were considered as candidates. These SNPs were subsequently clustered according to linkage disequilibrium (window size = 10,000 kb, r2 < 0.001), and weak instrumental variables (F statistic < 10) were excluded. Calculation of the F statistic strength: For each SNP included in the analysis, depending on the data situation, the following methods were used to calculate the R2 or F value. R2 = 2EAF(1-EAF)β2 or R2 = β2/(β2+SE2N), and F = R2(N-2)/(1-R2). Moreover, F ≥ 10 was required to evaluate the effectiveness of the instrument. The independence assumption was verified using MR-Egger regression.

#### 2.2.3 Sensitivity analysis

The P values for heterogeneity and horizontal pleiotropy were calculated. P > 0.5 indicated that there was no heterogeneity or pleiotropy in the samples. Any outliers were removed, and the MR-dependent estimates were re-evaluated. If the heterogeneity remained high after removing the outliers, a random effects model was used to assess the stability of the results. Finally, a leave-one-out analysis was conducted to verify the impact of each SNP on the overall causal relationship estimate.

##### 2.2.2.1 Batch screening of inflammatory factors and immune cells

1. Using 90 inflammatory factors as exposures and CH as the outcome, batch MR analysis was performed to screen positive inflammatory factors.
2. With 728 immune cells as exposures and CH as the outcome, batch MR analysis was carried out to screen positive immune cells.
3. Taking positive inflammatory factors as exposures and positive immune cells as outcomes, batch MR analysis was conducted to screen for double-positive inflammatory factors and double-positive immune cells.

##### 2.2.2.2 Batch mediation MR analysis

Taking the double-positive inflammatory factors as exposures, the double-positive immune cells as mediators, and CH as the outcome, mediation MR analysis was performed.

The analysis was carried out successively according to previously described methods (26). The analysis was performed in the following steps: (1) MR analysis was performed from exposure to outcome. (2) MR analysis was performed from outcome to exposure. (3) MR analysis was performed from exposure to mediator. (4) MR analysis was performed from mediator to outcome. In the first step, the total effect (beta_all) between the exposure and the outcome was evaluated. In the second step, we assessed whether there was a reverse effect of the outcome on the exposure. In the third step, the effect from the exposure to the mediator (beta1) was calculated. In the fourth step, the effect from the mediator to the outcome (beta2) was calculated, and the SNPs used in this step were different from those in the third step. The mediation effect was beta12 = beta1*beta2. The total effect could be decomposed into the direct effect of the exposure on the outcome (beta_dir) and the indirect effect mediated by the exposure through the mediator (beta12). The proportion of the mediation effect (Z) was calculated by dividing the indirect effect by the total effect, and the 95% confidence interval was calculated using the delta method.

##### 2.2.2.3 Batch mediation MR of IL-2

IL-2 was an inflammatory factor in the common enrichment pathway of the hub genes. The procedures were the same as those in part A. In this step, IL-2 was used as the exposure agent, 728 types of immune cells served as the mediators, and CH was regarded as the outcome.

##### 2.2.2.4 Screening of the immune pathways associated with the action of IL-2

1. With IL-2 as the exposure and 728 types of immune cells as the outcome, the positive immune cells affected by IL-2 were screened.
2. Taking the positive immune cells affected by IL-2 as the exposure and the double-positive inflammatory factors screened in part A as the outcome, the triple-positive inflammatory factors regulated by the positive immune cells affected by IL-2 were screened. In this way, the immune pathways through which IL-2 affects the outcome of CH were screened.

### 2.3 *In vitro* verification experiment

#### 2.3.1 Cell culture

##### 2.3.1.1 Source of experimental cells

This research protocol was reviewed and approved by the Ethics Committee of Gaomi Maternal and Child Health Hospital (Approval Number: 20230108-06). For research involving the use of human tissues, body fluids, or cell lines, written informed consent was obtained from the parents of the donors. The parents of the donors signed informed consent forms in accordance with the principles of the Declaration of Helsinki.

The thyroid cells used in this study were sourced from samples retained in our laboratory. Based on the diagnostic or intervention criteria for CH in most countries and regions, samples with a thyroid-stimulating hormone (TSH) level of 23 µIU/ml were classified as the CH group, and samples with a TSH level of 8 µIU/ml were classified as the normal group. The samples were obtained from two fetuses with missed abortion, among which the CH case had a gestational age of 34 + 2 weeks (G5P3), and the case in the normal group had a gestational age of 35 + 1 weeks (G3P2).The retained samples were residual thyroid tissue samples. A portion of each sample was crushed and then retained for further use in subsequent experiments. A portion of each sample was further isolated and purified from thyroid cells through the following steps.

##### 2.3.1.2 Isolation of thyroid cells

The thyroid tissue was isolated and rinsed with Hanks’ solution (YITA, Beijing, China), after which the visible blood vessels were removed. The rinsed thyroid tissue was placed in a petri dish and cut into small pieces until it reached a “minced meat” consistency. Three volumes of 0.25% collagenase (CellWorld, Beijing, China) were added to the Petri dish, and then the samples were digested at room temperature for 120 minutes. The digestion was stopped, the digestive fluid was aspirated, and then the samples were filtered through two layers of stainless-steel cell sieves with a mesh size of 100 μm. The sieves were then extensively rinsed, and the eluate was collected. The eluate was centrifuged at 1,000 r/s for 5 minutes at room temperature, and the supernatant was removed.

##### 2.3.1.3 Culture of thyroid cells

After centrifugation, the precipitate was added to DMEM containing 100×10³ U/L sodium heparin (Solarbio, Beijing, China), 20% fetal bovine serum (Optic, Shanghai, China), 10 mg/L bFGF (Sino Biological, Beijing, China), 100×10³ U/L penicillin, and streptomycin. The mixture was inoculated into a 24-well culture plate and then placed in an incubator at 37 °C with 5% CO_2_. The medium was replaced regularly during the culture period.

##### 2.3.1.4 Purification of thyroid cells

Five to six days after primary culture, if the number of cells was sufficiently small, the cells were isolated using the mechanical detachment method. The culture dish was observed under an inverted microscope. If the thyroid cells had grown into clonal aggregates, which looked like relatively flat and thin, resembling squamous epithelial cells, or cubic and columnar ones, marks were made on the bottom of the culture dish with a pen, and the thyroid cell clones were selected. Then, the other cells were scraped off with a cell scraper. Under low-power fields, 5 representative fields of view were selected (avoiding areas with excessively dense or sparse cells) and counted separately under a microscope. The target cells were thyroid cells. The number of target cells and the total number of cells were counted, and the cell purity (%) was calculated as (total number of target cells in all fields of view/total number of cells in all fields of view) * 100%.

##### 2.3.1.5 Subculture of thyroid cells

For cell culture, 0.125% trypsin (Nordic BioSite, Sweden) and fibronectin-coated culture dishes were used for screening and subculture. First, the thyroid cells were digested with a low concentration of trypsin so that they could adhere more quickly to the culture dish containing fibronectin. Trypsin (0.125%) was added, and the culture dish was subsequently shaken to ensure that the digestion mixture covered all the cells. The culture dish was observed under an inverted microscope for 4–5 minutes. The cells became round and wrinkled, the intercellular spaces increased, and most of the paving stone-shaped cells were nearly detached from the cell wall. The digestion was then terminated, the digestion mixture was removed, DMEM containing 10% FBS was added, and the medium was removed. The cells were repeatedly pipetted against the wall of the culture dish or plate. After dissociation, a cell suspension was formed and inoculated into the culture dish.

##### 2.3.1.6 Identification of thyroid cells

The obtained cells were sent for STR detection (**Supplementary Figure S1**). After confirming that the obtained cells were thyroid cells, the cells were then used for experiments.

#### 2.3.2 Plasmid constructs

The sequences of the hub genes were obtained from the National Center for Biotechnology Information (NCBI) (**Supplementary Table S2**). The primers were designed according to the coding DNA sequence (CDS) of the target genes (with the stop codon removed). The expression plasmids were constructed via vector double digestion and homologous recombination. To optimize gene expression, the G4S sequence GGTGGGGGCGGC was inserted between the two genes. Restriction enzyme cleavage sites were added to both ends of the primers, and 2–3 protective bases were randomly added to the ends. SnapGene was used to determine whether the number of base pairs after the fusion gene was spliced into the plasmid was a multiple of 3 to avoid translation mutations. If there were translation problems, bases needed to be randomly added to the primers to make the number of base pairs a multiple of 3 and offset the translation mutations. The plasmid pUC-18 (LMAI Bio, Shanghai, China) was selected as the expression vector (**Supplementary Figure S2**). Two restriction sites, HindIII and BamHI, were selected. The vector was double digested with HindIII (TaKaRa, Liaoning, China) and BamHI (Beyotime, Shanghai, China). The fragments were recovered after gel electrophoresis. Homologous arms and linkers were added to each target fragment by PCR, and the products were recovered after gel electrophoresis. Subsequently, homologous recombination was carried out among multiple fragments and the vector, and the four hub genes were ligated into the expression vector to construct the plasmids (**Supplementary Figure S3**). The ligation products were subsequently transformed into competent *Escherichia coli* DH5α (Whenzhou KeMiao Biological Technology Co., Ltd., Wenzhou, Zhejiang, China). The competent strains were cultured in medium without antibiotics, and some of the products were transferred to a Petri dish containing ampicillin. After single colonies grew on the plate, several colonies were randomly picked for qPCR identification after plasmid transfection into target cells to determine whether the hub genes could be successfully expressed in the target cells and to judge whether the plasmids had been successfully constructed.

#### 2.3.3 Grouping and plasmid transfection

The experimental groups were divided as follows: According to the TSH grouping criteria described previously, the CH group and the normal group were identified. Some of the samples from the CH group were collected, and after plasmid transfection, they formed the experimental group. For each group, 4,500 µl of 20% DMEM (Absin, Shanghai, China), 500 µl of fetal bovine serum (Optic), and 200 µl of penicillin plus streptomycin (P/S) (Absin) were added. First, cell resuspension, cell subculture, and cultivation were carried out. Cells in the logarithmic growth phase were selected, and the plasmid DNA was transfected using Lipofectamine 2000 (Invitrogen, Hangzhou, Zhejiang, China). The diluted plasmid DNA and Lipofectamine 2000 reagent were gently mixed and incubated at room temperature for 15–20 minutes to form a transfection complex. The transfection mixture was added dropwise to the culture container containing the cells, and the culture container was gently shaken to ensure that the complex was evenly distributed on the cell surface. The cell culture container was then returned to the cell incubator and cultured for an additional 6 hours, with three replicate wells in each group. An additional 20 transfection wells were selected, transfected according to the above steps, and subsequently used to determine the transfection effect by RT-qPCR for statistical analysis of transfection efficiency.

#### 2.3.4 RT–qPCR

Total RNA was extracted from the cells of each group, and the RNA was reverse transcribed to synthesize cDNA using specific primer sequences (**Supplementary Table S3**). Reverse transcription–quantitative polymerase chain reaction (RT–qPCR) was performed using a Gentier 96E fluorescence quantitative PCR instrument (TIANLONG, China). The data were analyzed for relative quantification using the 2-ΔΔCT method, and GAPDH was uniformly selected as the internal reference.

##### 2.3.4.1 Comparison of the expression of hub genes

RT–qPCR was used to analyze the expression of hub genes in the normal group and the CH group.

##### 2.3.4.2 Identification of the effect of plasmid transfection

The normal group and the experimental group were selected. After 3 days of culture, RT–qPCR was used to identify the expression of the hub genes in the two groups after plasmid transfection.

##### 2.3.4.3 Verification of the expression of STAT5 and MTORC1

All groups were selected, and thyroid tissues from the same source were added to each group to simulate the surrounding immune infiltration environment. After 4 days of culture, RT–qPCR was used to assess the expression of STAT5 and MTORC1 after plasmid transfection.

##### 2.3.4.4 Identification of genes related to cell activity

The CH group and the experimental group were selected, and thyroid tissues from the same source were added to each group to simulate the surrounding immune infiltration environment. After 4 days of culture, in accordance with previous methods (27), RT–qPCR was used to determine the expression of proliferating cell nuclear antigen (PCNA), B-cell lymphoma-2 (Bcl-2), and matrix metalloproteinase-2 (MMP-2). PCNA plays a key role in cell proliferation, is expressed mainly in the cell nucleus, and is closely related to DNA replication and cell cycle regulation (28). It is often used as a marker of cell proliferation and plays an important role in the growth and repair of normal tissues (29). Bcl-2 is an antiapoptotic protein located in the mitochondrial membrane and other sites, and it controls cell survival by regulating the intracellular apoptotic signaling pathway (30). MMP-2 is a member of the protease family that is secreted by cells and can degrade the components of the extracellular matrix (31). It participates in physiological processes such as tissue remodeling, can affect the survival of cells, and plays an important role in maintaining and regulating the stability of cell functions (32).

#### 2.3.5 Detection of cell proliferation by MTT assay

All groups were selected, and thyroid tissues from the same source were added to each group to simulate the surrounding immune infiltration environment. After 4 days of culture, the MTT cell proliferation assay was used to detect cell proliferation. A Cell Toxicity Detection Kit 500T (Wanlei Biotechnology, Shanghai, China) and a FlexA-200 Full-Wavelength Microplate Reader (Aosheng, Hangzhou, China) were used. As previously described (33), the MTT concentration was set at 0.1 mg/mL, and the transmission light wavelength was 565 nm. In a 96-well plate, 5,000 cells were seeded into each well.

#### 2.3.6 Effects of high expression of hub genes on inflammatory factors and free thyroxine

All groups were used, and thyroid tissues from the same source were added to each group to simulate the surrounding immune infiltration environment. Moreover, 10 μl of human thyrotropin-releasing hormone at a concentration of 200 μg/ml (Prospec, Israel) was added to each group. After 8 days of culture, a double-antibody sandwich ELISA was performed on the collected culture medium after treatment to detect the four inflammatory factors screened by MR and FT4. The culture medium samples were immediately centrifuged at 4,000 × g for 5 minutes, and the culture supernatants were collected and stored at −80 °C until use. In accordance with the protocol described by the manufacturer (see **Supplementary Figure S4** for reagent information and instructions), ELISA kits were used to measure the levels of IL-2, interleukin-18 (IL-18), osteoprotegerin (OPG), natural killer cell receptor 2B4 (CD244), and FT4. The measurement was carried out using a BioTek Epoch full-wavelength microplate reader (Epoch, USA). A standard curve was plotted according to the OD values and concentrations of each standard, and the concentrations of each sample were calculated on the basis of the standard curve and the OD values of each sample.

### 2.4 Statistical analysis

The statistical analysis software used was SPSS 18.0 and R (version 4.4.1). A value of P < 0.05 was considered statistically significant. The unpaired sample t test was used for comparisons between two groups, and the LSD t test was used for comparisons among multiple groups.

## 3 Results

### 3.1 Analysis of transcriptomic characteristics

**Figure 1:**
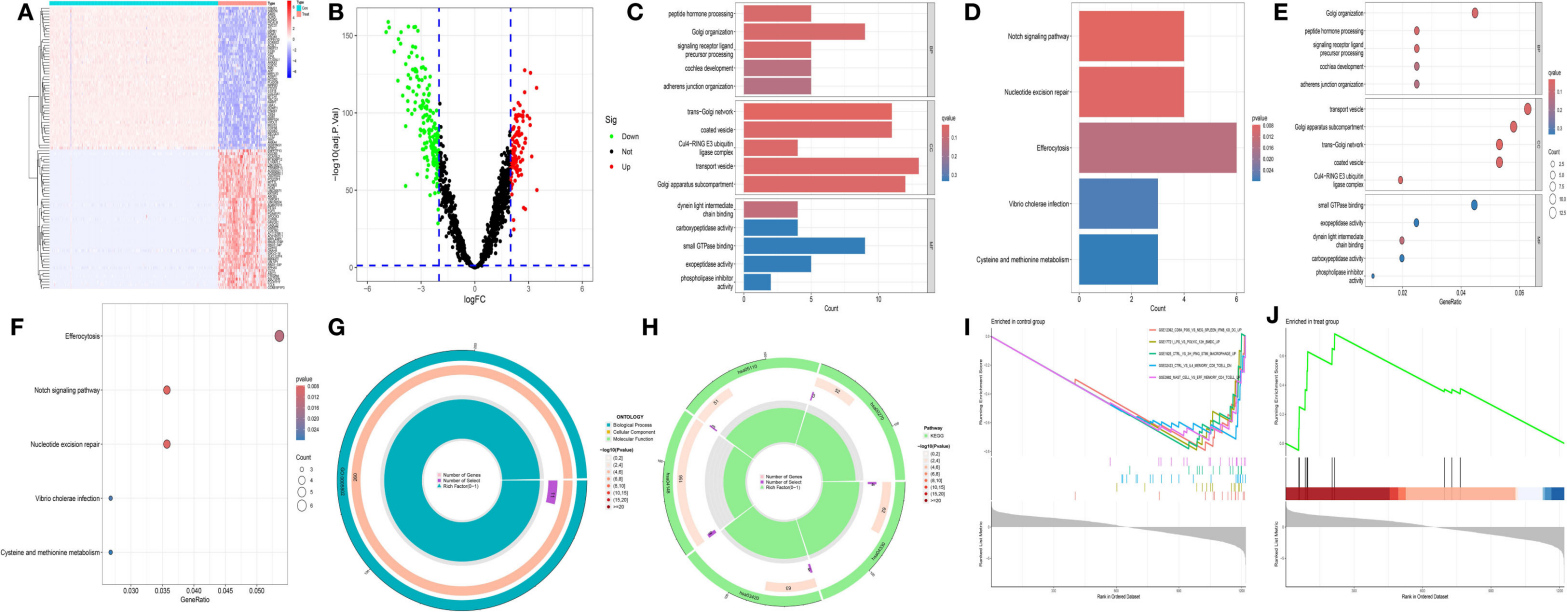
Preliminary analysis of transcriptomic data. **(A)** Heatmap showing the differentially expressed genes (DEGs) between CH and normal samples. The horizontal axis represents samples: blue (“con”) represents the normal group, and red (“Treat”) represents the CH group. The vertical axis represents genes. Red indicates high gene expression, and dark blue indicates low expression. **(B)** The volcano plot displays DEGs. Red, green, and black dots represent upregulated, downregulated, and nonsignificant genes, respectively, in the CH group compared with the normal group. **(C, D)** Bar charts of Gene Ontology (GO) and KEGG enrichment analyses: redder colors indicate greater differences, and bluer colors indicate smaller differences. The line length represents the number of enriched genes. **(E, F)** Bubble charts of the GO and KEGG analyses: a redder color indicates a greater significant difference, whereas a bluer color indicates a smaller difference. The bubble size represents the number of enriched genes. **(G, H)** Circular plots of the GO and KEGG analyses: the outermost circle is the GO ID, the inner circle is the number of enriched genes, followed by the number of enriched DEGs, and the innermost circle is the gene proportion. A redder color indicates more significant DEG enrichment. **(I, J)** Gene set enrichment analysis.

A total of 284 DEGs were identified between the CH group and the normal group, among which 110 genes were upregulated (log2FC > 2) and 174 genes were downregulated (log2FC < -2) (**Figures 1A, B**). Gene Ontology (GO) enrichment analysis revealed that the DEGs related to CH were significantly enriched in the functional network associated with the Golgi apparatus (**Figures 1C, E, G**). Kyoto Encyclopedia of Genes and Genomes (KEGG) enrichment analysis revealed that the DEGs related to CH were associated mainly with amino acid metabolism and bacterial infection (**Figures 1D, F, H**). Immune-related gene set enrichment analysis (GSEA) revealed that the number of active genes in the CH group was lower than that in the normal group, indicating that there were associations among CTRL (a protein derived from human blood and a novel biomarker), vaccines, and peripheral blood mononuclear cells (PBMCs) in CH (**Figures 1I, J**).

### 3.2 Screening of hub genes

**Figure 2:**
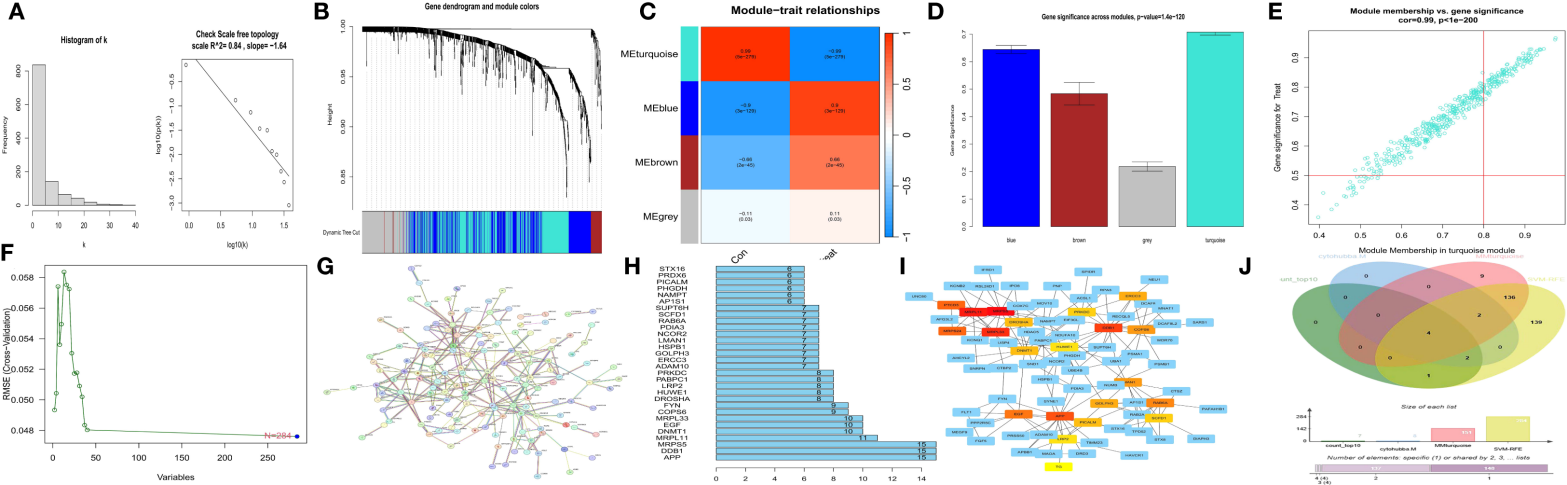
Screening of hub genes. **(A)** Scale-free network topological relationship diagram. **(B)** Weighted gene coexpression network analysis (WGCNA) coexpression network. **(C)** Heatmap of the correlation analysis between modules and clinical features. Red represents a positive correlation, and blue represents a negative correlation. **(D)** Gene importance graph. The horizontal axis represents the module name, and the vertical axis represents gene importance. **(E)** Scatter plot of the turquoise module. **(F)** Prediction of hub genes via support vector machine-recursive feature elimination (SVM-RFE). The horizontal axis represents the number of genes, and the vertical axis represents the error of cross-validation. **(G)** Protein–protein interaction (PPI) network. **(H)** Statistical graph of gene connection numbers. **(I)** Core network generated via the MCC algorithm of the cytoHubba plugin in Cytoscape. **(J)** Venn diagram of four characteristic gene sets.

We first observed that the correlation coefficient of the scale-free network topology diagram (**Figure 2A**) was 0.84, which was greater than the threshold of 0.8, proving that the selected power value could effectively construct a scale-free network. Correlation analysis between modules and clinical traits (MM) (**Figure 2C**) and gene significance analysis (GS) (**Figure 2D**) revealed that the turquoise module was the most dominant module. Characteristic genes with MM > 0.8 and GS > 0.5 were obtained from the scatter plot of the turquoise module (**Figure 2E**).

Second, using the JSVM-RFE machine learning algorithm, 284 characteristic genes at the minimum point of cross-validation were obtained (**Figure 2F**).

Third, by utilizing the protein–protein interaction (PPI) network (**Figure 2G**) and screening according to the number of gene connections, characteristic genes with more than 10 connections were determined (**Figure 2H**).

Fourth, according to the network interaction diagram (**Figure 2I**) drawn by the MCC algorithm of cytoHubba in Cytoscape, the top 20 characteristic genes characterized by this algorithm were selected.

An intersection operation was subsequently performed on the above four gene sets (**Figure 2J**), and finally, APP, DDB1, MRPS5, and MRPL33 were identified as hub genes.

### 3.3 Verification of hub genes and GSEA of coordinated genes

**Figure 3:**
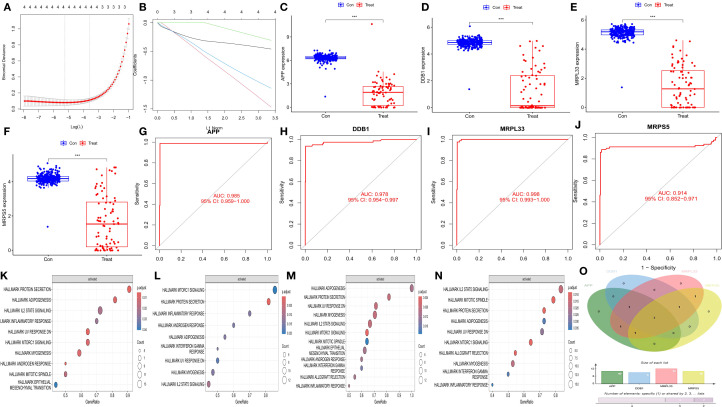
Validation of hub genes and GSEA enrichment analysis of synergistic genes. **(A)** Cross-validation plot of the least absolute shrinkage and selection operator (LASSO) regression. The horizontal axis represents the logarithmic value of (λ), and the vertical axis represents the cross-validation error. **(B)** LASSO regression plot. **(C–F)** Differential box plots of four hub genes. The horizontal axis represents different groups, and the vertical axis represents the expression levels of the hub genes. **(G–J)** Receiver operating characteristic (ROC) curves of the four hub genes. The horizontal axis represents the false positive rate, expressed as (1 - specificity), and the vertical axis represents the true positive rate, expressed as sensitivity. **(K–N)** Gene set enrichment analysis (GSEA) of the synergistic genes of the four hub genes. **(O)** Venn diagram of the GSEA results of the four hub genes.

The reliability of the hub genes was confirmed by three methods: least absolute shrinkage and selection operator (LASSO) regression analysis (**Figures 3A, B**), box plot drawing (**Figures 3C–F**), and the area under the receiver operating characteristic curve (AUC) (**Figures 3G–J**). Through gene set enrichment analysis (GSEA) of the coordinated genes, the enrichment pathways related to each hub gene were determined (**Figures 3K–N**), and their common enrichment pathways (**Figure 3O**) were as follows: PROTEIN SECRETION, ADIPOGENESIS, IL2 STAT5 SIGNALING, INFLAMMATORY RESPONSE, UV RESPONSE DN, MTORC1 SIGNALING, and MYOGENESIS. The analysis revealed that the low expression of the hub genes activated signaling pathways such as the IL2, STAT5, and MTORC1 pathways.

### 3.4 Immune cell infiltration analysis

**Figure 4:**
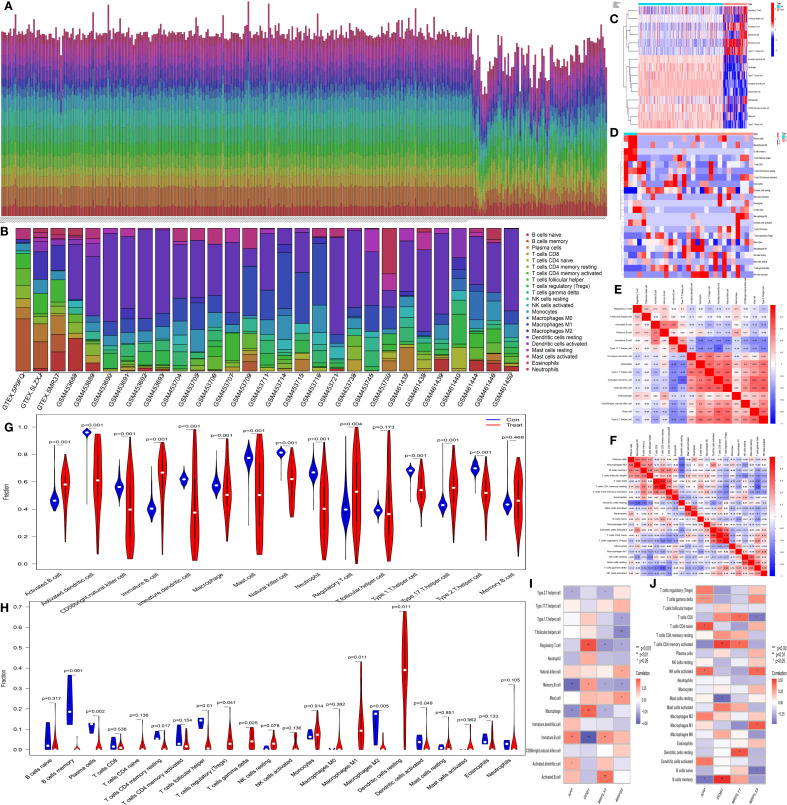
Analysis of immune cell infiltration. **(A, B)** Bar charts of data from single-sample gene set enrichment analysis (ssGSEA) related to immunity and CIBERSORT immune cell infiltration analysis. The horizontal axis represents the sample names, and the vertical axis represents the percentage of immune cells. **(C, D)** Heatmaps of the results of ssGSEA and CIBERSORT. The horizontal axis represents different samples, where blue (“con”) represents the normal group and red (“Treat”) represents the CH group. The vertical axis represents different immune cells. Red indicates high expression, and dark blue indicates low expression. **(E, F)** Heatmaps of the correlations between immune cells identified by ssGSEA and CIBERSORT. The redder the color is, the greater the positive correlation between the two cells; the bluer the color is, the greater the negative correlation between the two cells. **(G, H)** Violin plots of immune cells identified by ssGSEA and CIBERSORT. The horizontal axis represents immune cells, and the vertical axis represents the percentage of immune cells. Blue represents the normal group, and red represents the CH group. P represents the P value of the difference between the two groups. **(I, J)** Heatmaps of the correlation analysis of immune cells. The horizontal axis represents gene names, and the vertical axis represents immune cell names. The redder the color is, the greater the positive correlation between the two cells; the bluer the color is, the greater the negative correlation between the two cells. *P < 0.05, **P < 0.01, ***P < 0.001.

The immune-related single-sample gene set enrichment analysis (ssGSEA) and CIBERSORT immune cell infiltration analysis presented similar results. The histograms of immune cell infiltration (ssGSEA: **Figure 4A** and CIBERSORT: **Figure 4B**) revealed that there was little difference in the composition of immune cells among the samples. In addition, the heatmaps (ssGSEA: **Figure 4C** and CIBERSORT: **Figure 4D**) revealed obvious differences in the content and composition of immune cells between the normal group and the CH group, whereas the relevant heatmaps (ssGSEA: **Figure 4E** and CIBERSORT: **Figure 4F**) more easily revealed the degree of correlation among different types of immune cells. The violin plots (ssGSEA: **Figure 4G** and CIBERSORT: **Figure 4H**) demonstrated that there were differences in the content and composition of most immune cells between the CH group and the normal group. The heatmaps from the immune cell correlation analysis (ssGSEA: **Figure 4I** and CIBERSORT: **Figure 4J**) confirmed that all the hub genes were closely related to immune cells.

### 3.5 Batch MR analysis

#### 3.5.1 Batch screening of inflammatory factors and immune cells

Through batch MR analysis of 90 types of inflammatory factors, a total of 8 inflammatory factors were identified (**Supplementary Table S4** - infla). For the batch MR analysis of 728 types of immune cells, 13 immune cells were identified (**Supplementary Table S4** - imm). There were 19 regulatory pathways in total, through which inflammatory factors exerted their effects on CH, with immune cells acting as mediators (**Supplementary Table S4** - relationship). The indicators, including heterogeneity (**Supplementary Table S4** - heterogeneity), pleiotropy (**Supplementary Table S4** - pleiotropy), the SNP status of all the exposure data described above (**Supplementary Table S4** - SNPS), the results of 5 MR calculation methods (**Supplementary Table S4** - OR), the results of single-SNP analysis (**Supplementary Table S4** - single-pOR), and parameters such as beta_all, beta1, beta2, beta_dir, Z, and 95% confidence intervals of each pathway (**Supplementary Table S4** - effect), all met the requirements. The forest plots, funnel plots, scatter plots, and leave-one-out forest plots for the four-step screening of 20 immune pathways are shown in **Supplementary Figure S5** [A - S].

#### 3.5.2 Batch mediation MR of IL-2

IL-2 was identified to directly promote the progression of CH, with (CD27 on IgD - CD38+ B cell) as the mediator (**Supplementary Table S4** - [IL-2 effect], **Supplementary Figure S5** - T).

#### 3.5.3 Screening of immune pathways affected by IL-2

First, a total of 19 positive immune cells affected by IL-2 were identified (**Supplementary Table S4**-[IL-2imm], **Supplementary Figure S5**-[U - m]).

Next, three corresponding relationships of triple-positive inflammatory factors regulated by positive immune cells affected by IL-2 were identified (**Supplementary Table S4**-[IL-2-imm-immfa], **Supplementary Figure S5**-[n-p]).

Third, IL-2 positively regulated (CD27 on IgD-CD38+ B cell) and (CD27 on switched memory B cell)/natural killer cell receptor 2B4 (CD244) and negatively regulated (CD33dim HLA DR+ CD11b - absolute count)/interleukin-1 (IL-18) and (CD28+ CD4-CD8-T-cell %T cell)/osteoprotegerin (OPG), thereby promoting the progression of CH.

### 3.6 Experimental validation

#### 3.6.1 Comparison of the expression of hub genes in the normal group and the CH group

**Figure 5:**
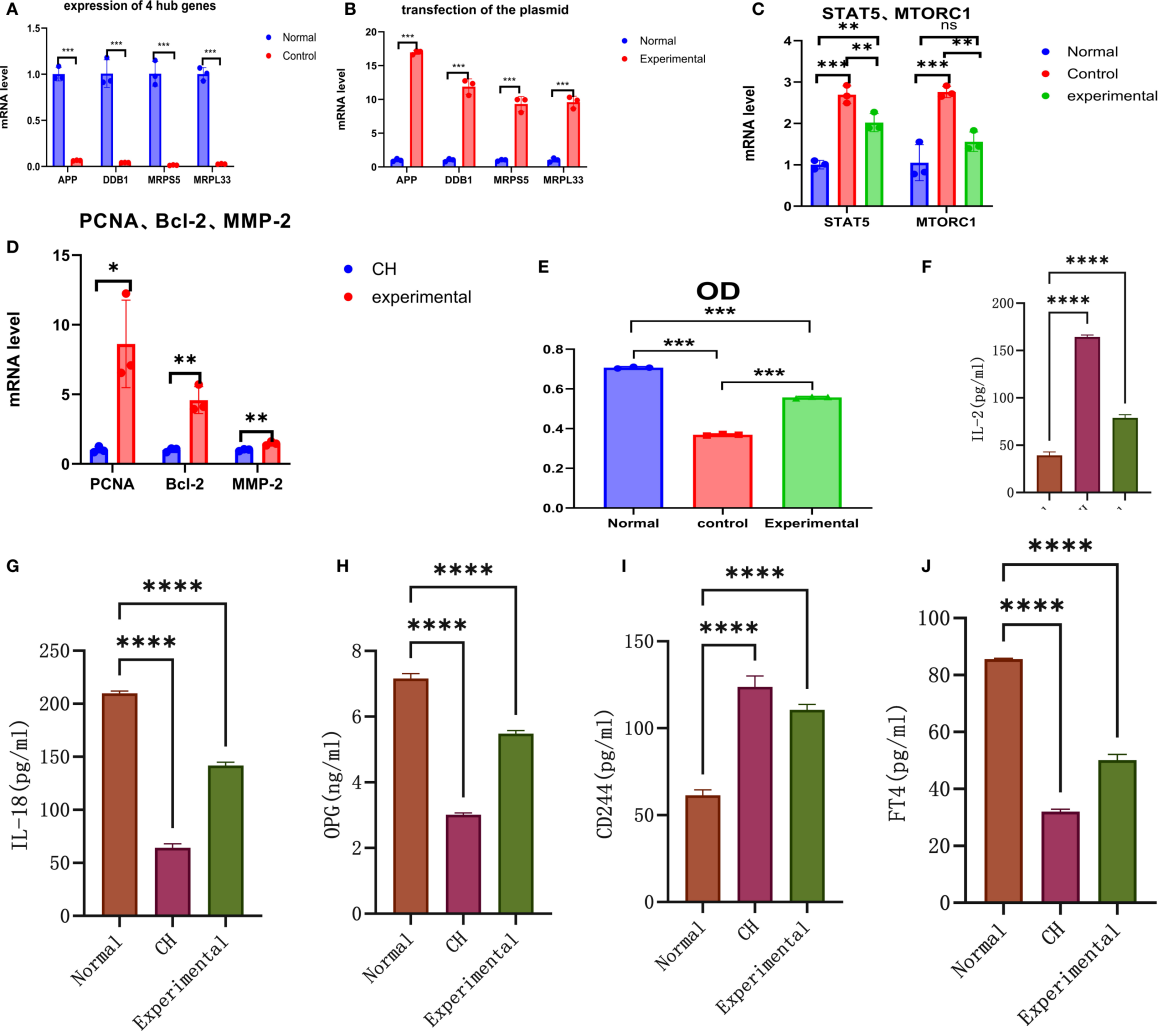
*In vitro* validation experiments. **(A)** Validation of the expression of the hub genes. **(B)** Validation of plasmid transfection. **(C)** Expression of STAT5 and MTORC1 in each group. **(D)** Expression of PCNA, Bcl-2, and MMP-2 in the CH group and the experimental group. **(E)** Comparison of cell proliferation in each group. **(F–J)** Expression diagrams of IL-2, IL-18, OPG, CD244, and FT4 in each group. *P < 0.05, **P < 0.01, ***P < 0.001, ****P < 0.0001.

The purity of the cells we used was 94.7%.APP, DDB1, MRPS5, and MRPL33 were expressed at low levels in CH (**Table 1**, **Supplementary Table S5**-verified, **Figure 5A**), which was consistent with the bioinformatics results.

**Table 1:** RT-qPCR verified the expression of 4 hub genes in the two groups of cells.

Gene	Cohort 1				Cohort 2			
	Normal group	CH group	t	*P*	Normal group	CH group	t	*P*
APP	1.002 ± 0.073	0.065 ± 0.004	22.199	0.000	1.009 ± 0.159	0.059 ± 0.015	10.300	0.001
DDB1	1.007 ± 0.150	0.042 ± 0.001	11.143	0.000	1.007 ± 0.146	0.075 ± 0.039	10.675	0.000
MRPS5	1.006 ± 0.134	0.014 ± 0.003	12.819	0.000	1.003 ± 0.087	0.043 ± 0.010	19.053	0.000

#### 3.6.2 Identification of plasmid transfection

The CH cells transfected with the plasmids highly expressed the four hub genes (**Table 2**, **Supplementary Table S5**-transfection, **Figure 5B**), indicating successful transfection. The transfection efficiency was 90%.

**Table 2:** The transfection of the plasmid verified by RT–qPCR.

Gene	Normal Group	Experimental group	t	*P*
APP	1.014 ± 0.212	16.960 ± 0.307	-74.029	0.000
DDB1	1.018 ± 0.229	11.890 ± 1.145	-16.127	0.000
MRPS5	1.005 ± 0.122	9.324 ± 1.103	-12.984	0.000
MRPL33	1.032 ± 0.307	9.591 ± 0.885	-15.826	0.000

#### 3.6.3 Verification of the expression of STAT5 and MTORC1

The expression of STAT5 and MTORC1 was greater in CH cells than in normal cells. After the hub genes were highly expressed, the expression levels of STAT5 and MTORC1 tended to decrease but were still higher than the normal levels overall (**Table 3**, **Supplementary Table S5**-STAT5 and MTORC1, **Figure 5C**).

**Table 3:** Conduct the verification of the expression situations of STAT5 and MTORC1.

Gene	Group	Cohort 1					Cohort 2				
		Mean 1	Mean 2	S1	S2	*P*	Mean 1	Mean 2	S1	S2	*P*
STAT5	Normal vs. CH	1.004	2.694	0.103	0.214	0.000	1.026	5.108	0.271	1.028	0.000
	Normal vs. Experimental	1.004	2.021	0.103	0.219	0.001	1.026	2.158	0.271	0.403	0.079
	CH vs. Experimental	2.694	2.021	0.214	0.219	0.004	5.108	2.158	1.028	0.403	0.002
MTORC1	Normal vs. CH	1.053	2.762	0.434	0.136	0.000	1.006	5.144	0.138	0.733	0.000
	Normal vs. Experimental	1.053	1.559	0.434	0.238	0.082	1.006	2.770	0.138	0.358	0.004
	CH vs. Experimental	2.762	1.559	0.136	0.238	0.003	5.144	2.770	0.733	0.358	0.001

#### 3.6.4 Identification of genes related to cell activity

PCNA, Bcl-2, and MMP-2 were expressed at low levels in CH, demonstrating their relatively low activity. After the hub genes were highly expressed, the expression of PCNA, Bcl-2, and MMP-2 increased, indicating that the hub genes could increase the activity of thyroid cells (**Table 4**, **Supplementary Table S5**-Cell Activity, **Figure 5D**).

**Table 4:** Identification of genes related to cell activity.

Gene	Cohort 1				Cohort 2			
	CH	Experimental	t	*P*	CH	Experimental	t	*P*
PCNA	1.015 ± 0.221	8.622 ± 3.144	-4.180	0.014	1.006 ± 0.135	4.261 ± 0.610	-9.025	0.001
Bcl-2	1.008 ± 0.146	4.585 ± 0.970	-6.315	0.003	1.080 ± 0.503	4.955 ± 1.826	-3.545	0.024
MMP-2	1.002 ± 0.072	1.466 ± 0.145	-4.981	0.008	1.031 ± 0.298	5.392 ± 1.262	-5.826	0.004

### 3.7 Detection of cell proliferation by MTT assay

The expression of the four hub genes was positively correlated with cell proliferation (**Table 5**, **Supplementary Table S6**, **Figure 5E**). When the hub genes were highly expressed, the level of cell proliferation increased to a certain extent, but it was still lower than the normal level.

**Table 5:** OD values in each group.

Group (OD)	1	2	S1	S2	*P*
Normal vs. CH	0.707	0.369	0.005	0.004	0.000
Normal vs. Experimental	0.707	0.557	0.005	0.004	0.000
CH vs. Experimental	0.369	0.557	0.004	0.004	0.000

### 3.8 Effects of high expression of hub genes on inflammatory factors and FT4

The expression levels of each inflammatory factor and FT4 were calculated according to the ELISA standard curve (**Supplementary Table S7**).

First, the expression of IL-2 in the CH group was greater than that in the normal group. After the hub genes were highly expressed, the expression of IL-2 decreased, but it was still higher than the normal level (**Table 6**, **Figure 5F**).

**Table 6:** Effects of high expression of hub genes on inflammatory factors and FT4.

Category	Group	Mean 1	Mean 2	S1	S2	*P*
IL-2	Normal vs. CH	39.217	164.435	3.715	1.992	0.000
	Normal vs. Experimental	39.217	78.928	3.715	3.377	0.000
	CH vs. Experimental	164.435	78.928	1.992	3.377	0.000
IL-18	Normal vs. CH	209.917	64.202	1.967	3.785	0.000
	Normal vs. Experimental	209.917	141.702	1.967	3.221	0.000
	CH vs. Experimental	64.202	141.702	3.785	3.221	0.000
OPG	Normal vs. CH	7.163	3.015	0.145	0.052	0.000
	Normal vs. Experimental	7.163	5.480	0.145	0.099	0.000
	CH vs. Experimental	3.015	5.480	0.052	0.099	0.000
CD244	Normal vs. CH	61.449	123.756	3.085	6.265	0.000
	Normal vs. Experimental	61.449	110.552	3.085	3.132	0.011
	CH vs. Experimental	123.756	110.552	6.265	3.132	0.000
FT4	Normal vs. CH	85.604	32.006	0.282	0.884	0.000
	Normal vs. Experimental	85.604	50.137	0.282	1.970	0.000
	CH vs. Experimental	32.006	50.137	0.884	1.970	0.000

Second, the expression of both IL-18 and OPG in the CH group was lower than that in the normal group. After the hub genes were induced to be highly expressed, their expression increased but was still lower than normal. These results suggest that IL-2 was negatively correlated with IL-18 and OPG (**Table 6**, **Figure 5G**, **Figure 5H**).

Third, the expression of CD244 in CH was greater than normal. After the hub genes were highly expressed, CD244 expression decreased slightly, suggesting that IL-2 was positively correlated with CD244 (**Table 6**, **Figure 5I**). The above results were all consistent with the MR results.

Finally, the expression of FT4 in the CH group was lower than that in the normal group. After the hub genes were highly expressed, their expression increased but was still lower than the normal level (**Table 6**, **Figure 5J**).

## 4 Discussion

In this study, through bioinformatics analysis, we screened and validated four hub genes, namely, APP, DDB1, MRPS5, and MRPL33. The low expression of these genes is an important factor contributing to the pathogenesis of CH and is significantly correlated with immunity. The low expression of these genes promotes the expression of IL-2. Batch MR screening revealed that IL-2 promotes the progression of CH by positively regulating (CD27 on IgD-CD38+ B cells) and (CD27 on switched memory B cells)/CD244 and negatively regulating (CD33dim HLA DR+ CD11b-absolute count)/IL-18 and (CD28+ CD4-CD8-T-cell %T cell)/OPG. *In vitro* validation demonstrated that high expression of hub genes can lead to a decrease in IL-2 expression. Through the STAT5 and MTORC1 pathways and related immune cells, the expression of IL-18 and OPG subsequently increases, and the expression of CD244 decreases. These changes ultimately enhance the activity of thyroid cells and the synthesis of FT4.

APP, the amyloid precursor protein gene, is crucial for maintaining the normal function of the nervous system (34, 35). Studies have shown that abnormal functions of APP can lead to abnormal expression of inflammatory factors and complements (36, 37). Another study directly demonstrated that altered expression of thyroid APP may induce changes in thyroid epithelial cells and cell damage, thus contributing to the occurrence of thyroid autoimmune-related diseases (38). These findings are consistent with the results of our study. DDB1, damage-specific DNA-binding protein 1, plays important roles in DNA repair, replication, transcription, and transcriptional regulation of genes (39). DDB1 has been shown to directly regulate the cell cycle (40), and multiple studies have reported its close association with immunity (41, 42). These studies further corroborate our results. MRPS5, mitochondrial ribosomal protein S5, is an important component of the small subunit of the mitochondrial ribosome and is considered a regulator of mitochondrial mRNA translation (43–45). MRPS5is also associated with immunity (44). The observations linking DDB1 and MRPS5 with immunity are consistent with our results. The MRPL33 gene encodes a large mitochondrial subunit protein that may be involved in mitochondrial translation (46) and has functions similar to those of MRPS5. However, there is a lack of research on its association with immunity. Nevertheless, on the basis of the properties of mitochondria (47, 48), they are presumed to be closely related to the immune system. Additionally, few studies have directly investigated the relationship between DDB1, MRPS5, MRPL33 and CH. Our study, however, rationally links CH and immunity through these genes.

The GO enrichment analysis demonstrated that the DEGs in CH patients were markedly enriched in the functional network associated with the Golgi apparatus, which confirms that our findings are in accordance with the disease characteristics of CH (49). The KEGG enrichment analysis indicated that the DEGs in CH patients were predominantly related to amino acid metabolism and bacterial infection, which is consistent with studies conducted by other groups (50). On the other hand, GSEA revealed that the majority of genes in CH patients were in a suppressed state, which is in line with the low expression state of the hub genes that we screened.

GSEA of coordinated genes identified the common pathways of action of hub genes, such as the IL2, STAT5, and MTORC1 pathways. Numerous studies have confirmed that the pathogenesis of CH is related to immunity (23, 51). The STAT5 (52, 53) and MTORC1 (54, 55) pathways are essential for immune regulation. In this study, immune infiltration analysis further confirmed the close association between hub genes and immunity and that CH is accompanied by significant immune alterations, which is consistent with the results of previous studies (56). These observations are in accordance with our results highlighting the IL2, STAT5, and MTORC1 pathways as important links connecting hub genes and immunity. Subsequently, through batch MR, we screened immune cells affected by IL-2 and related inflammatory factors and preliminarily constructed an immunoregulatory network that promotes the progression of CH, with IL-2 as the starting point.

The MR results confirmed that IL-2 can act on specific subtypes of T and B cells to negatively regulate IL-18 and OPG and positively regulate CD244. The results of the *in vitro* validation experiments were consistent with the MR results and further confirmed that the hub genes were expressed at low levels in CH and activated the IL-2, STAT5, and MTORC1 pathways. These pathways, on the one hand, inhibited IL-18 and OPG and, on the other hand, activated CD244. High expression of these hub genes increased the activity of thyroid cells and promoted the synthesis of FT4 through the abovementioned pathways. Numerous studies have confirmed that IL-2 can regulate the STAT5 (57–59) and MTORC1 (60–63) pathways, which is consistent with our findings and further clarifies the upstream and downstream relationships. IL-2 deficiency has been reported in neonates, which is closely associated with abnormal regulation of tyrosine phosphorylation (64).One study noted that IL-2 is an identified susceptibility gene locus associated with autoimmune thyroid diseases (65). Another study demonstrated that low-dose IL-2 can induce a sharp decrease in the levels of thyroglobulin antibody (TG-Ab) and thyroid peroxidase antibody (TPO-Ab), thereby playing a certain role in the treatment of autoimmune thyroid diseases (66), which is consistent with the conclusions of our research. Low-dose IL-2 has the advantage of selectively promoting Treg cells while inhibiting effector T cells (such as Tfh and Th17 cells) to improve immune homeostasis (67). Its function has been confirmed in the treatment of autoimmune diseases, which is consistent with the conclusions of this study (68–70). Due to the lack of relevant equipment, the *in vitro* experiments did not verify the immune cells involved in MR, which is a limitation of this study. However, the research of other groups has helped to fill this gap to a certain extent. Previous studies indicate that STAT5 can regulate T cells (53, 71) and B cells (72, 73) and that MTORC1 can also regulate T cells (74, 75) and B cells (76, 77).Polymorphisms in regulatory T cell-related genes have been shown to modulate inflammatory responses in viral hepatitis (78), suggesting that similar variations in immune pathways may contribute to the pathogenesis of CH.

IL-18 is involved in the host’s defense against infections, stimulating both innate and adaptive immune responses. It acts on T helper 1 (Th1) cells, macrophages, natural killer (NK) cells, natural killer T (NKT) cells, B cells, and dendritic cells (DCs), and in the presence of IL-12, it leads to the production of interferon γ (IFN-γ) (79–81). In combination with our study, the KEGG enrichment analysis results indicated that bacterial infection is associated with CH, and the relatively low expression level of IL-18 in CH may increase the vulnerability of children with CH to infections by pathogens such as bacteria. Osteoprotegerin (OPG) is a secreted glycoprotein that functions as a decoy receptor for receptor activator of nuclear factor kappa-B ligand (RANKL) and plays a central role in bone turnover. It has been associated with many bone-related diseases (82). It is expressed in various organs and serves as an important marker for fibrosis and calcification (83). Under basal conditions, OPG is released by endothelial cells upon stimulation by inflammatory cytokines, hormones, and various circulating compounds (84). Previous studies have reported an association between CH and calcification (85, 86), which is consistent with our results. Cluster of differentiation 244 (CD244) is an activating receptor of NK cells with specific and signal transduction mechanisms. NK cells can eliminate virus-infected cells and tumor cells (87). Our study revealed that NK cells are in a relatively active state in CH, resulting in a greater ability to resist viruses. Thus, these results indicate that CH may be less affected by viruses.

However, our study has certain limitations. First, the small sample size may result in some heterogeneity in the conclusions of this study, though we were able to mitigate this to a certain extent by increasing the number of experimental batches. Additionally, we lack validation of immune cells, and further confirmation can be conducted via flow cytometry or IHC in the future.

Our study provides relevant evidence for medical professionals in the diagnosis and treatment of congenital hypothyroidism (CH), supporting inflammation-related screening. After identifying abnormalities in inflammatory markers such as interleukin-2 (IL-2), IL-2-targeted diagnostic and therapeutic interventions can be implemented. The reference ranges of thyroid hormones vary worldwide, which hinders the introduction of universal detection recommendations. According to current guidelines, levothyroxine treatment for preterm infants is administered only after a confirmed diagnosis (16). Currently, the diagnosis of CH relies mainly on neonatal disease screening (NBS). However, the distribution of thyroid-stimulating hormone (TSH) in NBS is continuous, and the cutoff values used have significantly decreased over time, with substantial differences among different jurisdictions (88). As a result, an increasing number of regions must resort to imaging as an auxiliary means to improve the detection rate of CH (89). Our study, therefore, provides new evidence for recommendations for immunological diagnosis and treatment related to CH.

## 5 Conclusion

**Figure 6:**
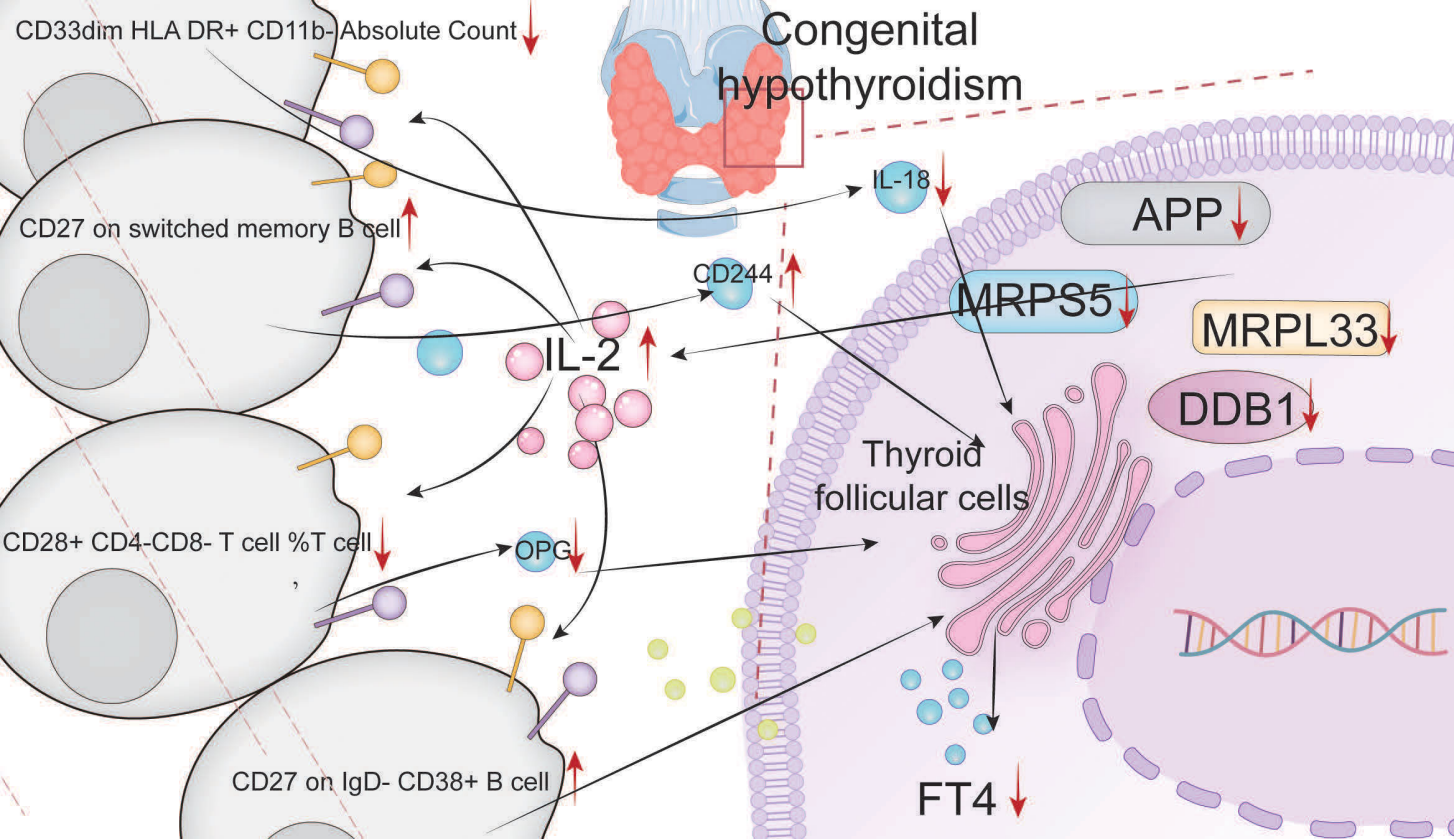
Pathogenic mechanism diagram of the immune regulation network of the hub genes.

APP, DDB1, MRPS5, and MRPL33 are hub genes with low expression levels in congenital hypothyroidism (CH). They can promote the expression of IL-2 and may positively regulate (CD27 on IgD-CD38+ B cells) and (CD27 on switched memory B cells)/CD244 via the STAT5 and MTORC1 pathways, while negatively regulating (CD33dim HLA-DR+ CD11b- Absolute Count)/IL-18 and (CD28+ CD4- CD8- T cells %T cells)/OPG, thereby facilitating the progression of CH (**Figure 6**).

## Data Availability

The original contributions presented in the study are included in the article/**Supplementary Material**. Further inquiries can be directed to the corresponding author.
