# Supplementary figures and images for: Identification of hub genes in congenital hypothyroidism and construction of the associated immune regulatory network
Source: Front Immunol. 2025 Oct 15;16:1608098. doi: 10.3389/fimmu.2025.1608098 (PMC12568518; doi:10.3389/fimmu.2025.1608098)

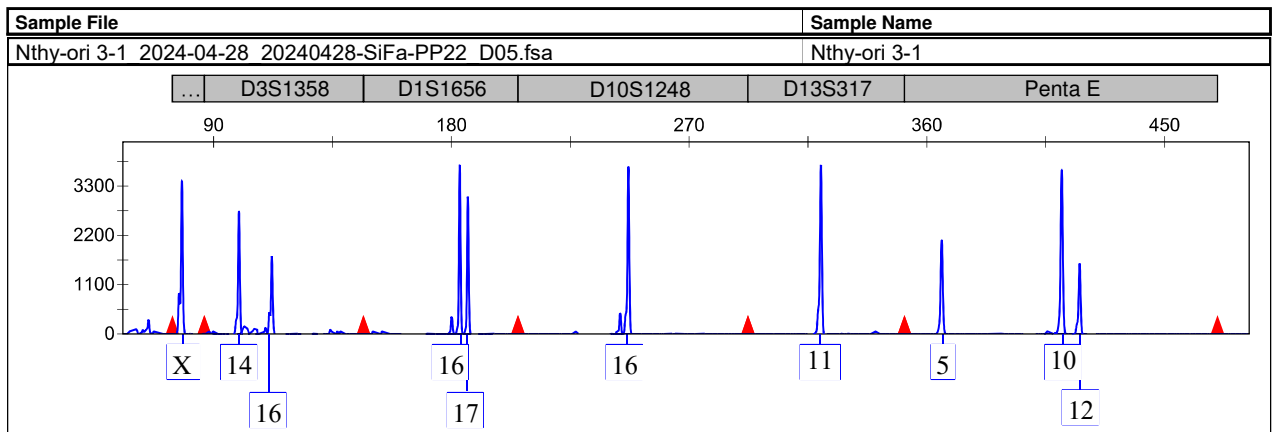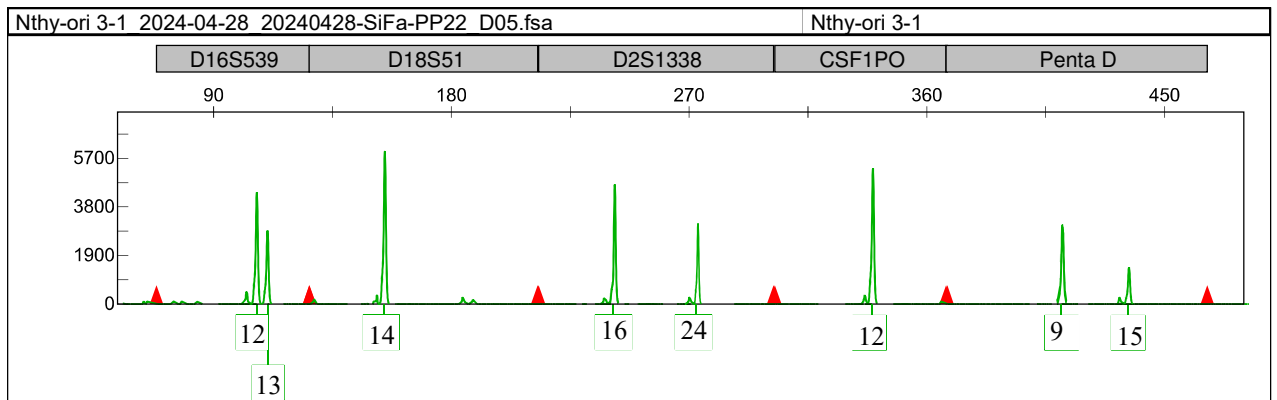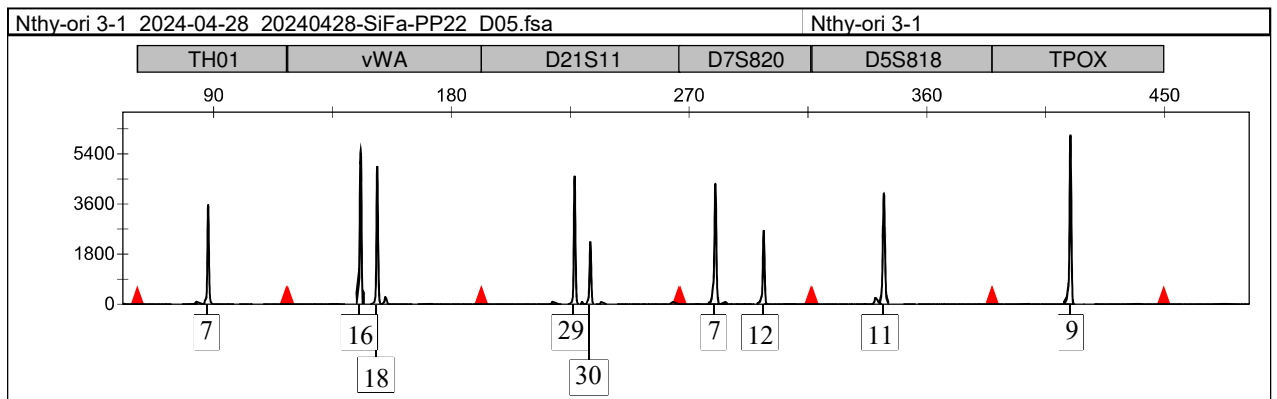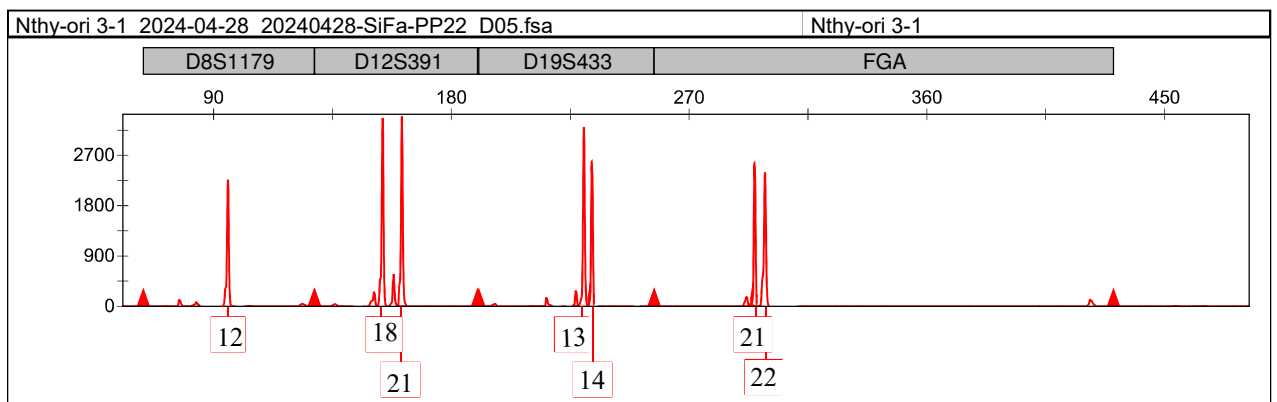

Supplement: Supplementary file 1 [file DataSheet1.pdf]

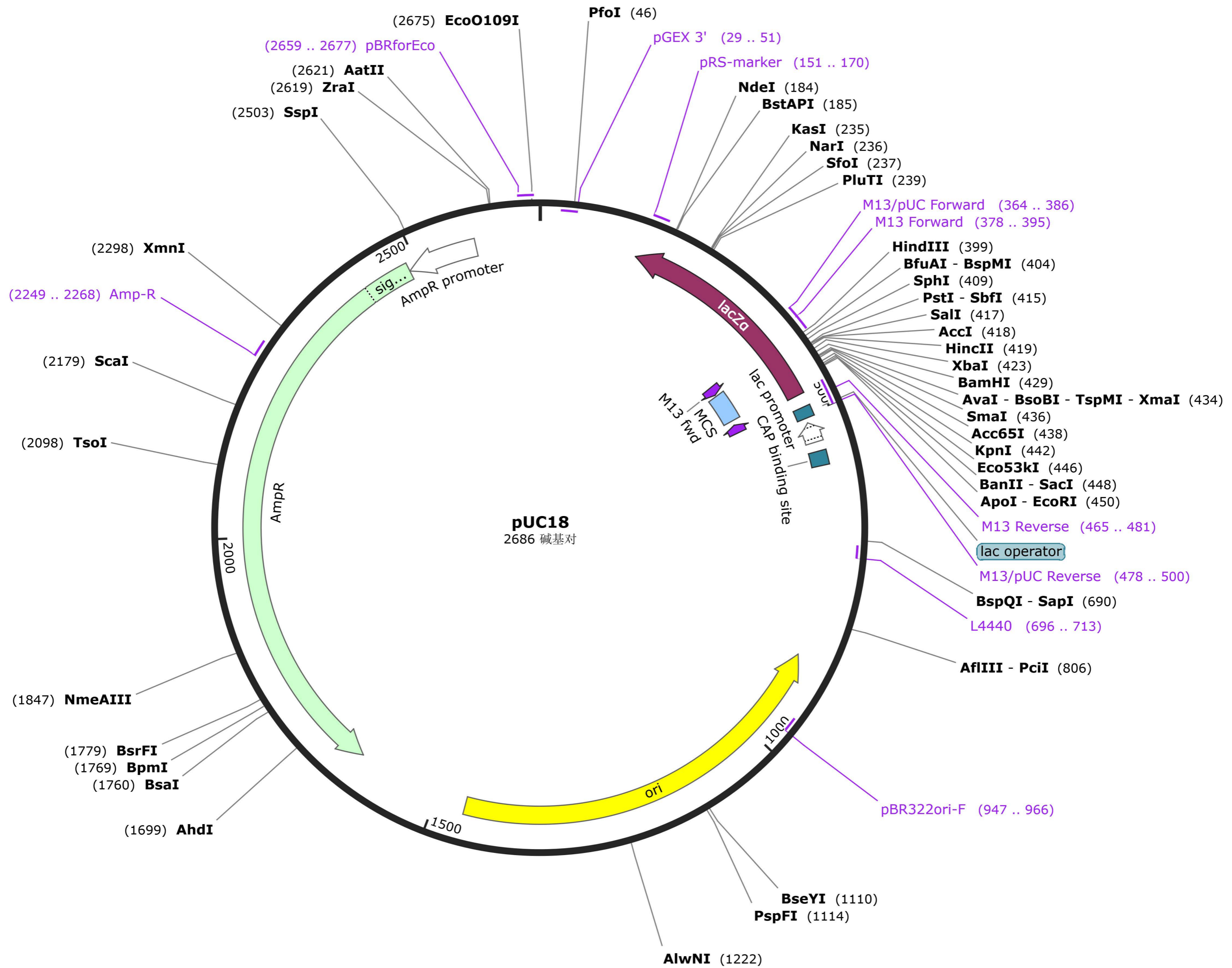

Supplement: Supplementary file 2 [file DataSheet2.pdf]

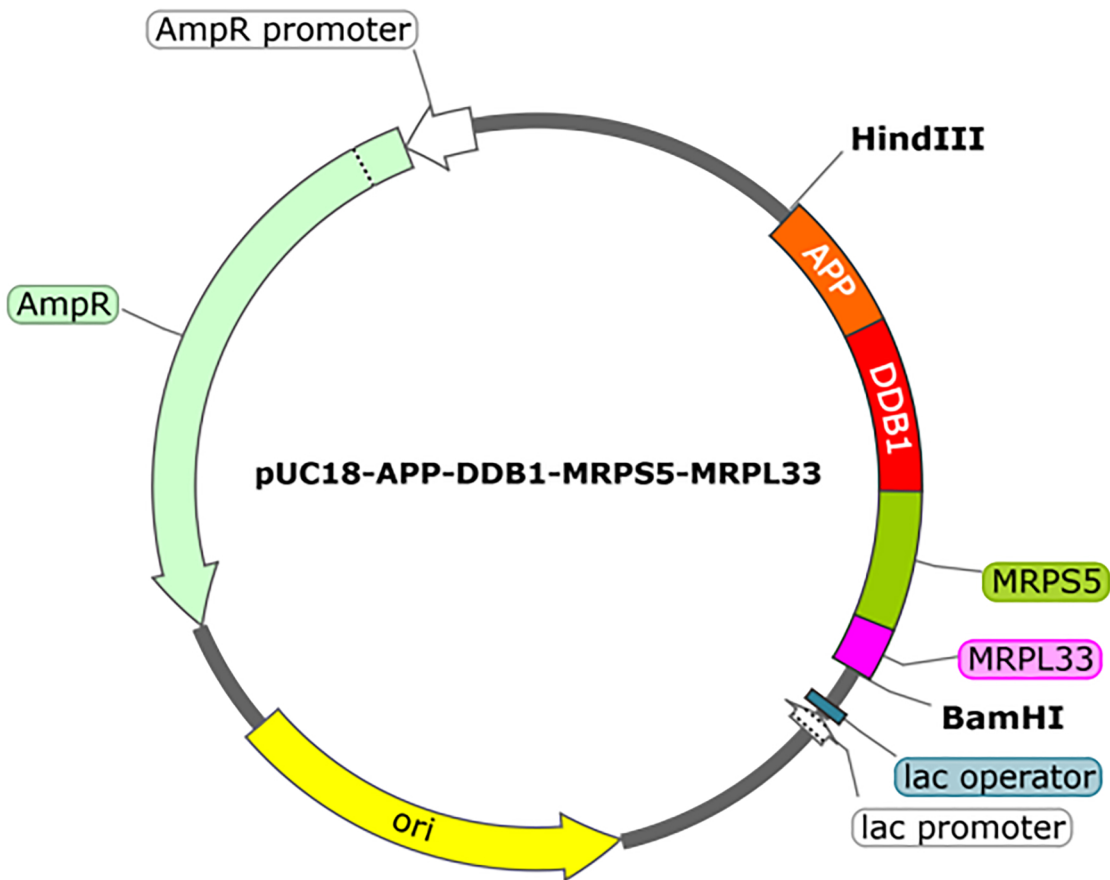

Supplement: Supplementary file 3 [file DataSheet3.pdf]

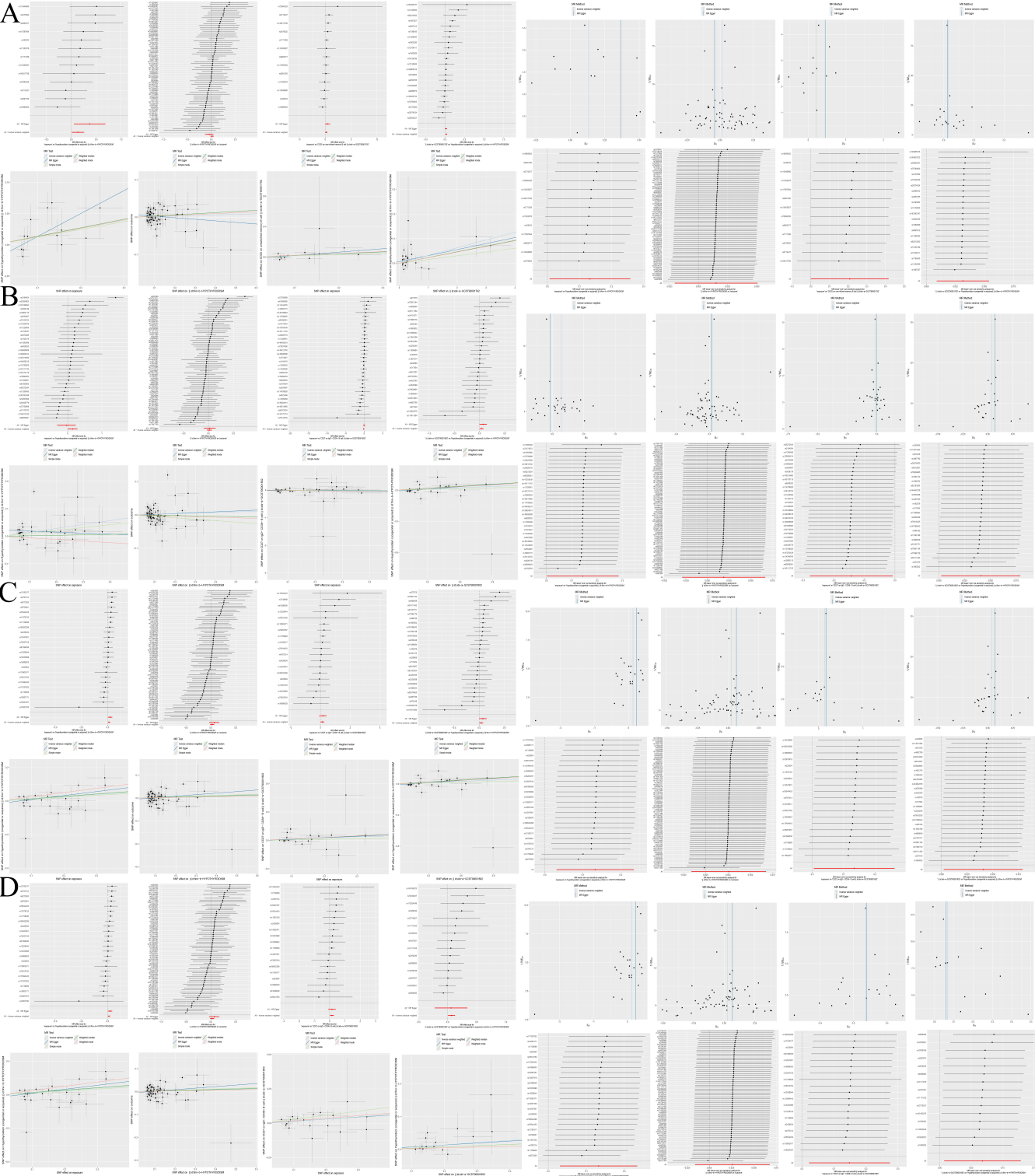

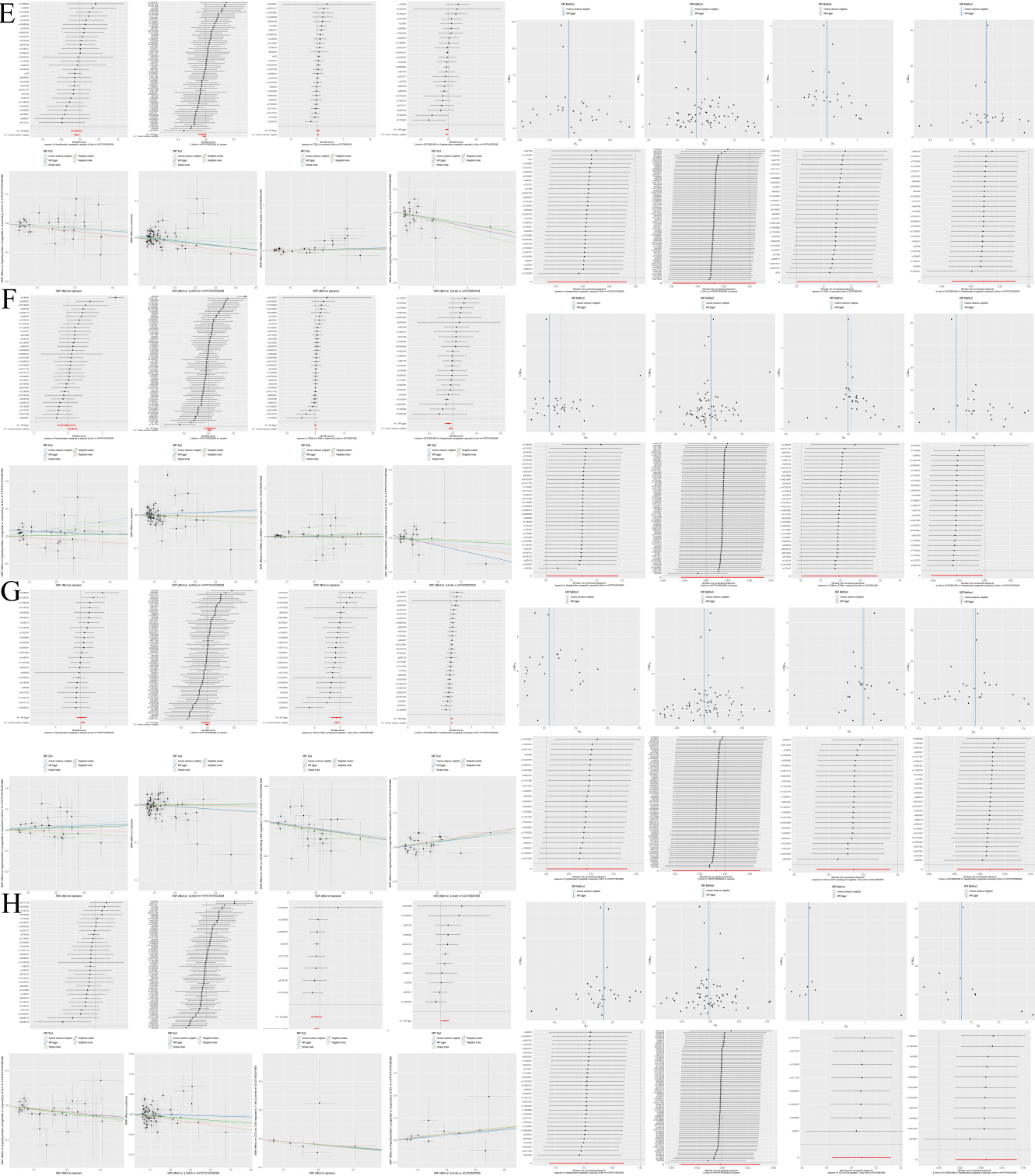



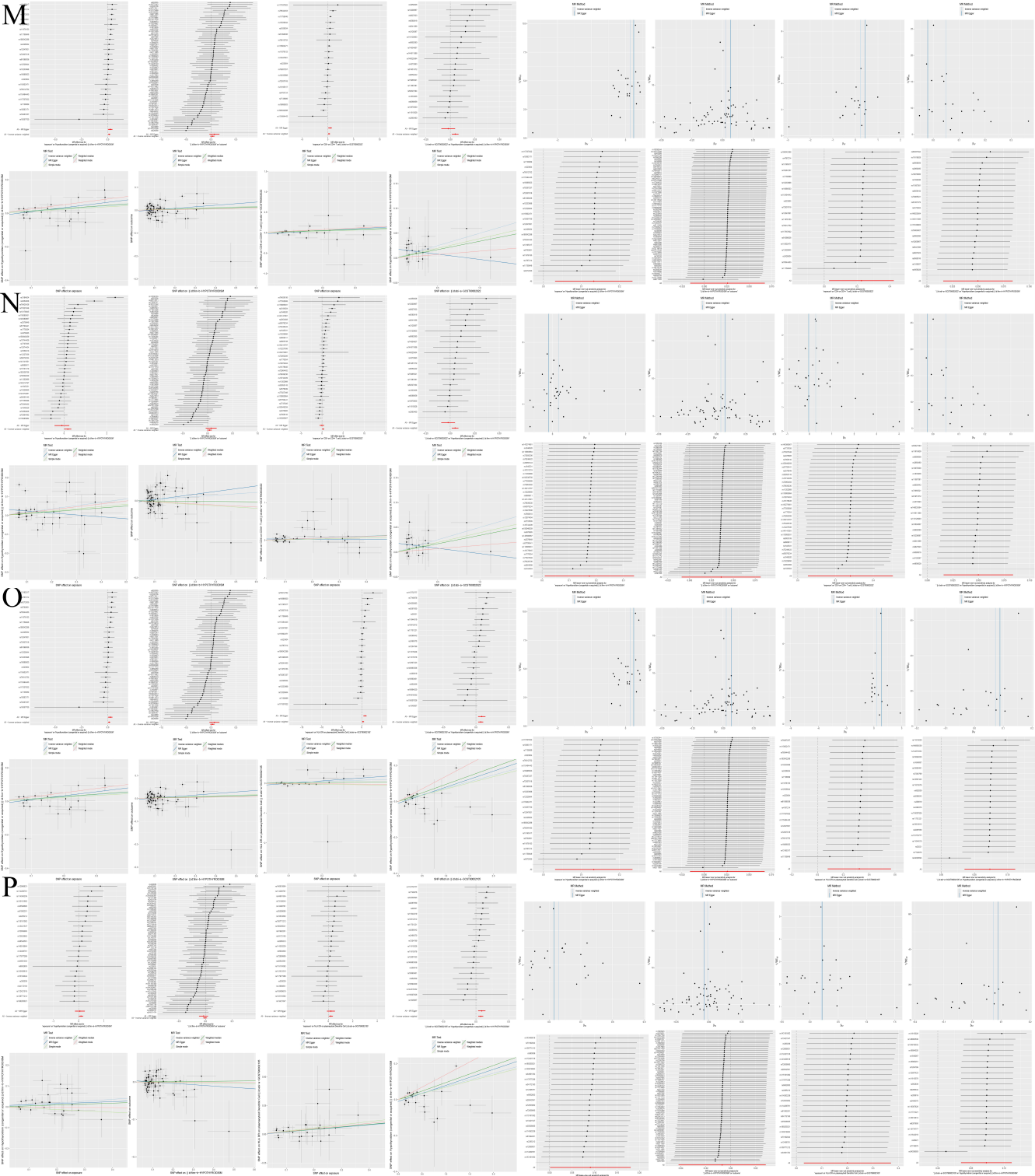



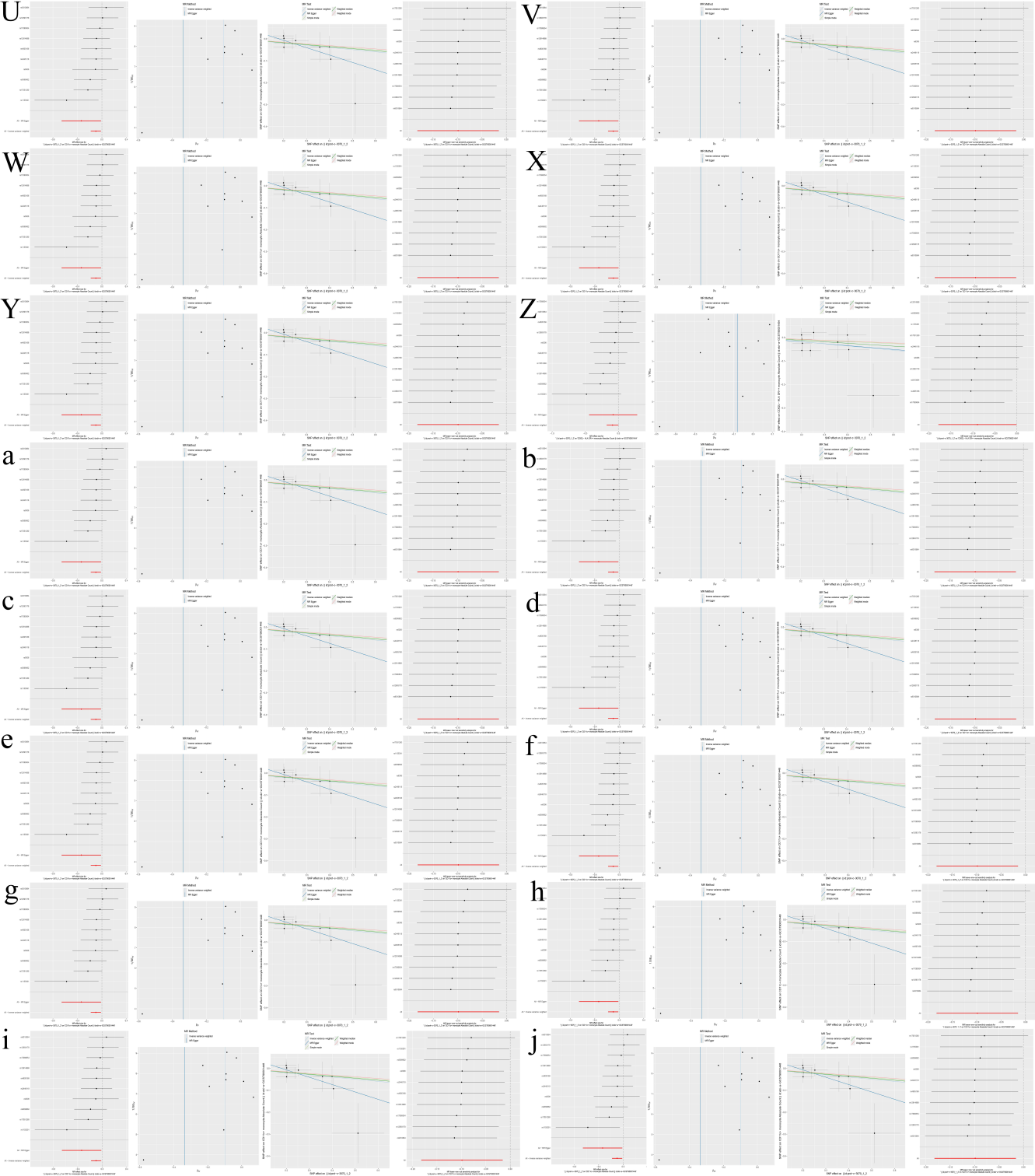

O

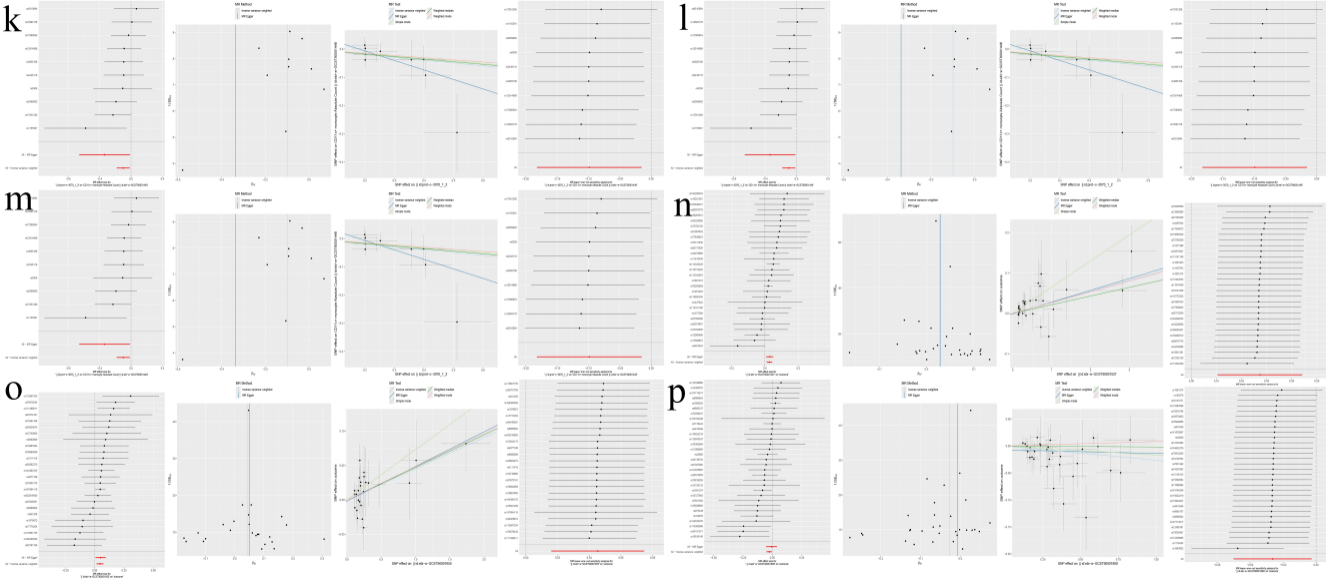

Supplement: Supplementary file 5 [file DataSheet5.pdf]
