# Supplementary material for: Identification of hub genes in congenital hypothyroidism and construction of the associated immune regulatory network
Source: Front Immunol. 2025 Oct 15;16:1608098. doi: 10.3389/fimmu.2025.1608098 (PMC12568518; doi:10.3389/fimmu.2025.1608098)
Supplement: Supplementary file 4 [file DataSheet4.pdf]

Beyotime

Support: +86-400-1683301 / info-intl@beyotime.com
Orders: order-intl@beyotime.com
Tel: +86-400-1683301 / +86-800-8283301
Add: 1500 Xinfei Rd, Songjiang, Shanghai, China
Web: www.beyotime.com

# Human IL-2 ELISA Kit

| Cat. No. | Product Name | Pack Size |
|---|---|---|
| PI580 | Human IL-2 ELISA Kit | 96T |

## Description:

- Beyotime's Human IL-2 ELISA Kit (Human Interleukin-2 Enzyme-Linked ImmunoSorbent Assay Kit) provides a specific and highly sensitive method for quantification of IL-2 in human serum, plasma, and cell culture supernatant.
- This product has high detection sensitivity, high specificity and good reproducibility. Multiple repeated assays show that the minimum detection amount is 16.5pg/ml, and there is no cross-reactivity with human IL-2sRα, IL-2sR/Fc Chimera, IL-2 sRβ, IL-2 sRγ, mouse IL-2, rat IL-2, etc. The intra-plate and inter-plate coefficients of variation are both less than 10%.
- Interleukin-2 (IL-2), also known as T cell growth factor (TCGF), is a glycosylated α-helical polypeptide with a molecular weight of approximately 15-18 kDa, belonging to the γc cytokine family and existing as a monomer with an extremely short half-life (<30 min). Mature human IL-2 comprises 133 amino acid residues with the 20-aa signal peptide removed. Its structural domain has an α-helical structure in its natural state, with post-translational modifications including O-glycosylation of the third Thr site and disulfide bond formation. There are three cysteine residues (Cys) at positions 58, 105 and 125. The disulfide bonds formed between cysteine residues at positions 58 and 105 is prerequisite for protein activation. Mature human IL-2 has 73%, 66%, 78% and 97% amino acid homology with canine, rat, cat and monkey IL-2, respectively. Although the amino acid homology between human IL-2 and mouse IL-2 is only 60%, human IL-2 can activate the IL-2 receptor on mouse cells. IL-2 is mainly produced by γδT cells, activated $CD4^+$ and $CD8^+$ T cells, neuronal cells, microglia and hematopoietic stem cells.
- The IL-2 receptor (IL-2R) is a heterotrimer comprising a 55 kDa CD25/IL-2 Rα chain, a 70 kDa IL-2 Rβ and a 65 kDa γc chain. IL-2 first binds to IL-2 Rα to form a dimeric complex and then recruits IL-2 Rβ and γc to form a tetrameric signaling complex. In addition to IL-2, IL-2 Rβ is also a component of the IL-15 tetrameric signaling complex, while γc is a common chain shared by IL-4, IL-7, IL-9, IL-15 and IL-21 receptors.
- IL-2 plays an important role in the cell proliferation of T cells, natural killer cells and B cells at different stages of response induced by antibody stimulation. Additionally IL-2 regulates the expression of gamma interferon, major histocompatibility antigens, stimulates the proliferation and differentiation of activated B cells, increases the activity of natural killer cells and inhibits the proliferation of granulocytes/macrophages. IL-2 also induces the proliferation and differentiation of oligodendrocytes.
- IL-2 binds to its receptor and activates the JAK-STAT signaling pathway through phosphorylation to deliver signals to the nucleus for regulation of target genes. In addition, IL-2 can also activate various downstream signaling pathways such as Ras/Raf/MEK/MAPK and PI3K/Akt/P70S6K by forming Grb2/SOS complexes under the action of Shc protein.
- This kit employs the double-antibody sandwich ELISA for quantification analysis of human IL-2 in samples (Figure 1). The monoclonal antibodies against human IL-2 are precoated on the plate as capture antibodies and when a standard or sample is added, the human IL-2 binds to the capture antibody. The biotin-conjugated human IL-2 antibody is then added and binds to human IL-2 on the plate to form a sandwiched immune complex. After that, HRP-labeled Streptavidin is added and binds to the sandwich immune complex through the specific interaction between biotin and streptavidin. Finally, the chromogenic reaction is initiated by the addition of TMB Solution. TMB produces a deep blue color during the enzymatic degradation of hydrogen peroxide by HRP, and the addition of Stop Solution gives a clear yellow color that absorbs at 450nm. The A450 value is directly proportional to the concentration of human IL-2 in samples which can be calculated from the standard curve generated in the same assay.

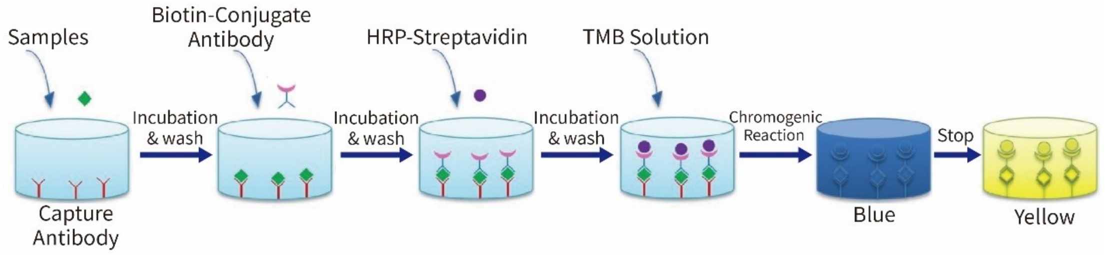


**Figure 1.** Schematic diagram of double-antibody sandwich ELISA

➢ This kit is sufficient for 96 assays.

## Packing List:

| Item | Component | Quantity |
|---|---|---|
| PI575-1 | 96-well Strip-well Plate Coated with Mouse IL-2 Antibody | 8 wells × 12 strips |
| PI575-2 | Assay Buffer | 5ml |
| PI575-3 | Standard Dilution Buffer | 10ml |
| PI575-4 | Mouse IL-2 Standard | 2-4 bottles |
| PI575-5 | Biotin-conjugated Mouse IL-2 Antibody | 10ml |
| PI575-6 | HRP-labeled Streptavidin | 10ml |
| PI575-7 | Wash Buffer (20X) | 30ml |
| PI575-8 | TMB Solution | 10ml |
| PI575-9 | Stop Solution | 5ml |
| PI575-10 | Adhesive Films (transparent) | 2 |
| PI575-11 | Adhesive Films (white) | 2 |
| Manual | — | 1 copy |

## Storage Conditions:

Store the Standard at 4ºC for up to 1-2 weeks, or -20ºC for up to 6 months. Store the other components in this kit at 4ºC for up to 6 months.

## Precautions:

➢ The standard is generally lyophilized powder. Check the instructions labeled on the standard vial to prepare the standard solution.

➢ Crystal precipitation may exist in Wash Buffer (20X) at low temperatures. Please dissolve it completely using a water bath at room temperature prior to use.

➢ Standard solution should be prepared freshly. Discard the rest after use.

➢ Avoid oxidizer and metal contamination that cause the invalidation of TMB Solution.

➢ Change pipette tips between different samples and liquids to prevent contamination and incorrect loading volumes.

➢ Do not mix or interchange reagents from different kit lots.

➢ It is particularly important to perform sufficient mixing of reactions to ensure an accurate result. Please shake the 96-well plate gently after the addition of reagents.

➢ Most procedures of this experiment should be performed at room temperature (25-28ºC). Temperature lower than 25ºC will result in a significant decrease in the absorbance value of reactions.

➢ The washing process is very important. Insufficient wash will result in reduced accuracy and increased experimental errors.

➢ Run all standards, controls, and samples in duplicate.

➢ Avoid the formation of air bubbles when adding the sample.

➢ This product is for R&D only. Not for drug, household, or other uses.

➢ For your safety and health, please wear a lab coat and disposable gloves during the operation.

## Instructions for Use:

1. **Preparation of the sample.**

   a. **Cell supernatant:** Centrifuge cell cultures at 100-500×g for 5 minutes to collect the supernatant.

   b. **Serum:** Leave the whole blood undisturbed at room temperature for 30 minutes to 2 hours. After the whole blood clots, collect the yellow supernatant (serum) by centrifuging the whole blood at 1000-2000×g for 10 minutes at 4ºC, and keep the serum on ice.

   ***Note:*** *Do Not add any preservatives or anticoagulants to serum.*

c. **Plasma:** Add heparin or EDTA anticoagulant to whole blood and place on ice after mix. After centrifuging at 1000-2000×g for 10 minutes at 4ºC, collect the yellow supernatant (plasma) and keep it on ice.

*Note 1: If the samples cannot be analyzed immediately, make aliquots and store them at -20ºC or -80ºC. Avoid repeated freeze-thaw.*

*Note 2: Samples should be clear and transparent. Remove any particulates from samples by centrifuging before being analyzed.*

*Note 3: Do not use hemolyzed, hyperlipidemia, or contaminated samples for analysis.*

*Note 4: Serum or plasma samples may need to be properly diluted with Assay Buffer before the assay.*

**2. Preparation of the kit.**

a. Thaw the reagents and equilibrate to room temperature (25-28ºC) prior to use. Store the reagents at 4ºC immediately after use.

b. Prepare an appropriate amount of 1X Wash Buffer by diluting the Wash Buffer (20X) with $ddH_2O$.

c. Reconstitute Standard to 1000pg/ml with Standard Dilution Buffer according to the instructions labeled on the standard vial. Mix gently and incubate for 15min at room temperature. Gently pipette the contents several times to dissolve the standard completely. Generally, each concentration of standard is analyzed at least in duplicates (at least 2 wells) and 100μl is needed for each well. If one bottle of Standard is not enough, use more bottles of Standard, but the contents from different bottles need to be mixed before performing dilutions.

d. Add 250μl of Standard Dilution Buffer to each of 5 tubes and make serial dilutions as shown in Figure 2 to obtain 5 concentrations: 1/2 (500pg/ml), 1/4 (250pg/ml), 1/8 (125pg/ml), 1/16 (62.5pg/ml), 1/32 (31.25pg/ml). Mix thoroughly between steps.

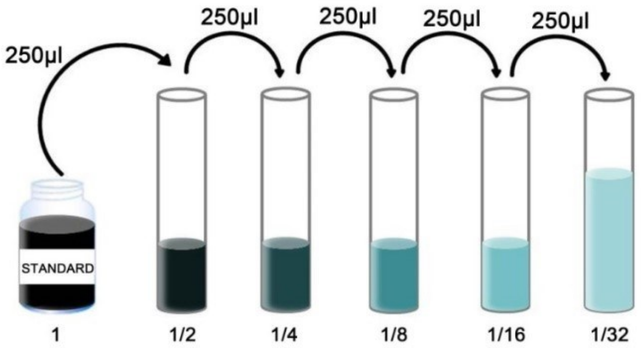


**Figure 2.** Dilution diagram for preparation of different concentrations of Standard.

**3. Perform ELISA.**

a. Determine the number of pre-coated 8-well strips required for the experiment. Insert the strips in a frame for use. Re-bag any unused strips and store them at 4ºC.

b. Prepare the Standard freshly and plot a standard curve for every run. Set up the blank control by only adding TMB Solution and Stop Solution.

c. Add 100μl of samples or standards to each well. Cover wells with Adhesive Films (transparent), and incubate for 2 hours at room temperature.

***Note:** For the assay of serum or plasma samples, add 50 μl of Assay Buffer followed by 50 μl of sample. If the dilution factor is large, add equal amounts of sample and Assay Buffer, and supplement the shortage to 100 μl with Standard Dilution Buffer. It is necessary to record the dilution factor of the sample.*

d. Thoroughly aspirate the solution by gently lowering a pipette tip into the bottom of each well and wash wells 5 times with 300μl 1X Wash Buffer. Allow the buffer to stand for 15-30 seconds before aspiration. After the last wash, invert the strip and tap dry on absorbent tissue.

e. Add 100μl of Biotin-conjugated Antibody to each well (***Note:** the antibody can be used directly without dilution*). Cover wells with Adhesive Films (transparent) and incubate for 1 hour at room temperature.

f. Thoroughly aspirate the solution and wash wells 5 times with 300μl of 1X Wash Buffer, as described in step 3d.

g. Add 100μl of HRP-labeled Streptavidin to each well (***Note:** the HRP-labeled Streptavidin can be used directly without dilution*), cover wells with Adhesive Films (white), and incubate at room temperature for 20 minutes in the dark. If the room temperature is low, prolong the incubation time as appropriate.

h. Thoroughly aspirate the solution and wash wells 5 times with 300μl of 1X Wash Buffer, as described in step 3d.

i. Add 100μl of TMB Solution to each well, cover wells with Adhesive Films (white), and incubate at room temperature for 15-20 minutes in the dark. If the room temperature is low, prolong the incubation time until the standard sample exhibits significant color change. If the concentration of target protein in sample is high, color change will occur soon.

j. Add 50μl of Stop Solution to each well. Read the absorbance at 450nm immediately after mixing.

**4. Analysis.**

a. Calculate the average A450 value for each standard and sample. Duplicates should be within 20 percent of the mean value.

b. Subtract the A450 value of blank control from the A450 values of standards and samples (This step can be omitted if there is no blank control).

c. Generate the standard curve by plotting the concentrations of standard on the abscissa and their corresponding A450 values on the ordinate (Figure 3).

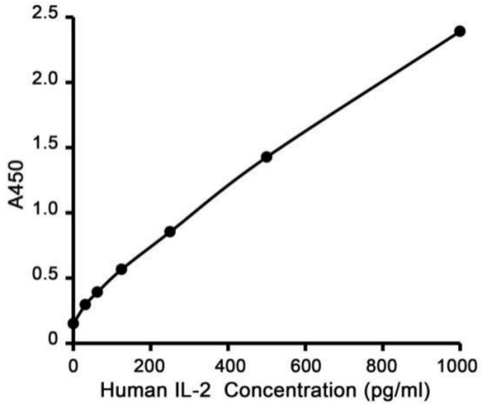


**Figure 3.** The standard curve of Beyotime's Human IL-2 ELISA Kit (PI580). This figure is for reference only, which may vary due to different experimental conditions.

d. Determine the concentrations of human IL-2 in samples from the standard curve based on their A450 values.

***Note:*** *Dilute samples producing signals greater than the upper limit of the standard curve in Standard Dilution Buffer and reanalyze. Multiply the concentration by the dilution factor.*

## Related Products:

| Cat.No. | Product Name | Package |
|---|---|---|
| PI301 | Mouse IL-1β ELISA Kit | 96T |
| PI303 | Rat IL-1β ELISA Kit | 96T |
| PI305 | Human IL-1β ELISA Kit | 96T |
| PI326 | Mouse IL-6 ELISA Kit | 96T |
| PI328 | Rat IL-6 ELISA Kit | 96T |
| PI330 | Human IL-6 ELISA Kit | 96T |
| PI508 | Mouse IFN-γ ELISA Kit | 96T |
| PI510 | Rat IFN-γ ELISA Kit | 96T |
| PI511 | Human IFN-γ ELISA Kit | 96T |
| PT512 | Mouse TNF-α ELISA Kit | 96T |
| PT516 | Rat TNF-α ELISA Kit | 96T |
| PT518 | Human TNF-α ELISA Kit | 96T |
| PI522 | Mouse IL-10 ELISA Kit | 96T |
| PI525 | Rat IL-10 ELISA Kit | 96T |
| PI528 | Human IL-10 ELISA Kit | 96T |
| PI575 | Mouse IL-2 ELISA Kit | 96T |
| PI577 | Rat IL-2 ELISA Kit | 96T |
| PI580 | Human IL-2 ELISA Kit | 96T |
| PI602 | Mouse Insulin ELISA Kit (Ultrasensitive) | 96T |
| PI606 | Rat Insulin ELISA Kit (Ultrasensitive) | 96T |

| | | |
|---|---|---|
| PI608 | Human Insulin ELISA Kit (Ultrasensitive) | 96T |
| PI612 | Mouse IL-4 ELISA Kit | 96T |
| PI615 | Rat IL-4 ELISA Kit | 96T |
| PI618 | Human IL-4 ELISA Kit | 96T |
| PI640 | Human IL-8 ELISA Kit | 96T |

Version 2021.11.23

Support: +86-400-1683301 / info-intl@beyotime.com
Orders: order-intl@beyotime.com
Tel: +86-400-1683301 / +86-800-8283301
Add: 1500 Xinfei Rd, Songjiang, Shanghai, China
Web: www.beyotime.com

# Human IL-18/IL-1F4 ELISA Kit

| Cat. No. | Product Name | Pack Size |
|---|---|---|
| PI558 | Human IL-18/IL-1F4 ELISA Kit | 96T |

## Description:

- Beyotime's Human IL-18 ELISA Kit (Human Interleukin-1β Enzyme-Linked ImmunoSorbent Assay Kit) provides a specific and highly sensitive method for quantification of IL-18 in human serum, plasma, and cell culture supernatant.
- This product has high detection sensitivity, high specificity and good reproducibility. Multiple repeated assays show that the minimum detection amount is 15.8 pg/ml and there is no cross-reactivity with human IFN-γ, IL-18 BPa, IL-18 Rα, mouse IL-18, and rat IL-18. The intra-plate and inter-plate coefficients of variation are both less than 10%.
- Interleukin-18 (IL-18), also known as IL-1F4, is a pro-inflammatory cytokine belonging to the IL-1 family that exhibits different immune effects in different environments. IL-18 is expressed mainly as a precursor protein with a molecular weight of 24 kDa in endothelial cells, epidermal cells, keratinocytes, γδ T cells, and phagocytes. The precursor IL-18 is activated intracellularly by caspase-1-regulated protein hydrolysis and is subsequently secreted as a mature IL18 of 17 kDa. Necrotic cells can also secrete the precursor IL-18 which is processed extracellularly by various proteases into mature IL-18. Activation of IL-18 occurs primarily upon infection or tissue injuries and promotes pathological damage in chronic inflammation.
- IL-18 has two receptors, IL-18 Rα and IL-18 Rβ. IL-18 first binds to IL-18 Rα and then recruits IL-18 Rβ to form a signaling receptor complex. IL-18 binding to IL-18 binding proteins exhibits opposite biological activities. In the presence of IL-12 or IL-15, IL-18 promotes the production of IFN-γ and induces cytolytic activity of $CD8^+$ T cells and natural killer cells, thereby enhancing the antiviral immune response. However, without IL-12 or IL-15, IL-18 promotes the production of Th2-type cytokines, IL-4 and IL-13. When co-existing with IL-1β or IL-23, IL-18 induces non-antigen-dependent production of IL-17. Meanwhile, IL-18 also promotes maturation of bone marrow-derived dendritic cells and induces a respiratory burst of neutrophils. In tumors, IL-18 has multiple biological activities, including enhancing antitumor immune responses, inhibiting or promoting neovascularization, and promoting tumor cell metastasis. The amino acid sequence of mature human IL-18 shares 63% similarity with mouse and rat IL-18.
- This kit employs the double-antibody sandwich ELISA for quantification analysis of human IL-18 in samples (Figure 1). The monoclonal antibodies against human IL-18 are precoated on the plate as capture antibodies and when a standard or sample is added, the human IL-18 binds to the capture antibody. The biotin-conjugated human IL-18 antibody is then added and binds to human IL-18 on the plate to form a sandwiched immune complex. After that, HRP-labeled Streptavidin is added and binds to the sandwich immune complex through the specific interaction between biotin and streptavidin. Finally, the chromogenic reaction is initiated by the addition of TMB Solution. TMB produces a deep blue color during the enzymatic degradation of hydrogen peroxide by HRP, and the addition of Stop Solution gives a clear yellow color that absorbs at 450nm. The A450 value is directly proportional to the concentration of human IL-18 in samples which can be calculated from the standard curve generated in the same assay.

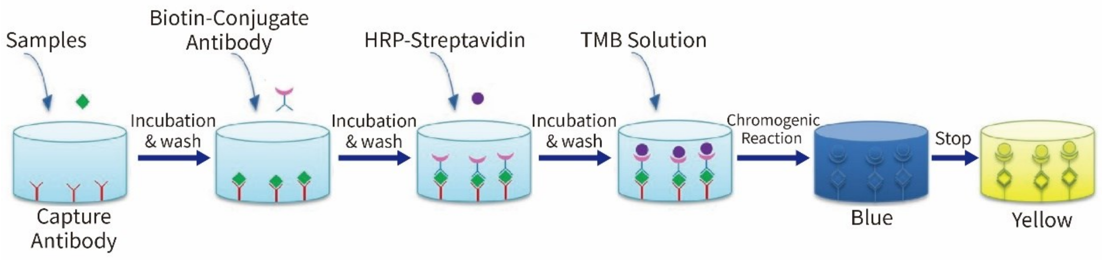


**Figure 1.** Schematic diagram of double-antibody sandwich ELISA

- This kit is sufficient for 96 assays.

## Packing List:

| Item | Component | Quantity |
|---|---|---|
| PI558-1 | 96-well Strip-well Plate Coated with Human IL- | 8 wells×12 strips |

| | | |
|---|---|---|
| | 18/IL-1F4 Antibody | |
| PI558-2 | Assay Buffer | 5ml |
| PI558-3 | Standard Dilution Buffer | 10ml |
| PI558-4 | Human IL-18/IL-1F4 Standard | 2-4 bottles |
| PI558-5 | Biotin-conjugated Human IL-18/IL-1F4 Antibody | 10ml |
| PI558-6 | HRP-labeled Streptavidin | 10ml |
| PI558-7 | Wash Buffer (20X) | 30ml |
| PI558-8 | TMB Solution | 10ml |
| PI558-9 | Stop Solution | 5ml |
| PI558-10 | Adhesive Films (transparent) | 2 |
| PI558-11 | Adhesive Films (white) | 2 |
| Manual | — | 1 copy |

## Storage Conditions:

Store the Standard at 4ºC for up to 1-2 weeks, or -20ºC for up to 6 months. Store the other components in this kit at 4ºC for up to 6 months.

## Precautions:

- The standard is generally lyophilized powder. Check the instructions labeled on the standard vial to prepare the standard solution.
- Crystal precipitation may exist in Wash Buffer (20X) at low temperatures. Please dissolve it completely using a water bath at room temperature prior to use.
- Standard solution should be prepared freshly. Discard the rest after use.
- Avoid oxidizer and metal contamination that cause the invalidation of TMB Solution.
- Change pipette tips between different samples and liquids to prevent contamination and incorrect loading volumes.
- Do not mix or interchange reagents from different kit lots.
- It is particularly important to perform sufficient mixing of reactions to ensure an accurate result. Please shake the 96-well plate gently after the addition of reagents.
- Most procedures of this experiment should be performed at room temperature (25-28ºC). Temperature lower than 25ºC will result in a significant decrease in the absorbance value of reactions.
- The washing process is very important. Insufficient wash will result in reduced accuracy and increased experimental errors.
- Run all standards, controls, and samples in duplicate.
- Avoid the formation of air bubbles when adding the sample.
- This product is for R&D only. Not for drug, household, or other uses.
- For your safety and health, please wear a lab coat and disposable gloves during the operation.

## Instructions for Use:

1. **Preparation of the sample.**

   a. **Cell supernatant:** Centrifuge cell cultures at 100-500×g for 5 minutes to collect the supernatant.

   b. **Serum:** Leave the whole blood undisturbed at room temperature for 30 minutes to 2 hours. After the whole blood clots, collect the yellow supernatant (serum) by centrifuging the whole blood at 1000-2000×g for 10 minutes at 4ºC, and keep the serum on ice.

   ***Note:*** *Do Not add any preservatives or anticoagulants to serum.*

   c. **Plasma:** Add heparin or EDTA anticoagulant to whole blood and place on ice after mix. After centrifuging at 1000-2000×g for 10 minutes at 4ºC, collect the yellow supernatant (plasma) and keep it on ice.

   ***Note 1:*** *If the samples cannot be analyzed immediately, make aliquots and store them at -20ºC or -80ºC. Avoid repeated freeze-thaw.*

   ***Note 2:*** *Samples should be clear and transparent. Remove any particulates from samples by centrifuging before being analyzed.*

   ***Note 3:*** *Do not use hemolyzed, hyperlipidemia, or contaminated samples for analysis.*

*Note 4:* *Serum or plasma samples may need to be properly diluted with Assay Buffer before the assay.*

2. **Reagent Preparation.**
   a. Thaw the reagents and equilibrate to room temperature (25-28ºC) prior to use. Store the reagents at 4ºC immediately after use.
   b. Prepare an appropriate amount of 1X Wash Buffer by diluting the Wash Buffer (20X) with $ddH_2O$.
   c. Reconstitute Standard to 750pg/ml with Standard Dilution Buffer according to the instructions labeled on the standard vial. Mix gently and incubate for 15min at room temperature. Gently pipette the contents several times to dissolve the standard completely. Generally, each concentration of standard is analyzed at least in duplicates (at least 2 wells) and 100μl is needed for each well. If one bottle of Standard is not enough, use more bottles of Standard, but the contents from different bottles need to be mixed before performing dilutions.
   d. Add 250μl of Standard Dilution Buffer to each of 5 tubes and make serial dilutions as shown in Figure 2 to obtain 5 concentrations: 1/2 (375pg/ml), 1/4 (187.5pg/ml), 1/8 (93.75pg/ml), 1/16 (46.875pg/ml), 1/32 (23.44pg/ml). Mix thoroughly between steps.

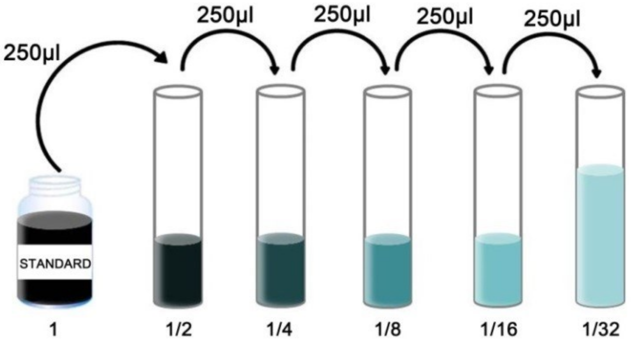


**Figure 2.** Dilution diagram for preparation of different concentrations of Standard.

3. **Perform ELISA.**
   a. Determine the number of pre-coated 8-well strips required for the experiment. Insert the strips in a frame for use. Re-bag any unused strips and store them at 4ºC.
   b. Prepare the Standard freshly and plot a standard curve for every run. Set up the blank control by only adding TMB Solution and Stop Solution.
   c. Add 100μl of samples or standards to each well. Cover wells with Adhesive Films (transparent), and incubate for 2 hours at room temperature.

      ***Note:*** *For the assay of serum or plasma samples, add 50 μl of Assay Buffer followed by 50 μl of sample. At this point, the sample is diluted 2 times. If the sample concentration is too high and beyond the detection range, add 50 μl of Assay Buffer first, and then add 50 μl of the diluted sample. The optimal dilution factor for cell supernatant samples needs to be determined by preliminary tests. It is necessary to record the dilution factor of the sample.*
   d. Thoroughly aspirate the solution by gently lowering a pipette tip into the bottom of each well and wash wells 5 times with 300μl 1X Wash Buffer. Allow the buffer to stand for 15-30 seconds before aspiration. After the last wash, invert the strip and tap dry on absorbent tissue.
   e. Add 100μl of Biotin-conjugated Antibody to each well (***Note:*** *the antibody can be used directly without dilution*). Cover wells with Adhesive Films (transparent) and incubate for 1 hour at room temperature.
   f. Thoroughly aspirate the solution and wash wells 5 times with 300μl of 1X Wash Buffer, as described in step 3d.
   g. Add 100μl of HRP-labeled Streptavidin to each well (***Note:*** *the HRP-labled Streptavidin can be used directly without dilution*), cover wells with Adhesive Films (white), and incubate at room temperature for 20 minutes in the dark. If the room temperature is low, prolong the incubation time as appropriate.
   h. Thoroughly aspirate the solution and wash wells 5 times with 300μl of 1X Wash Buffer, as described in step 3d.
   i. Add 100μl of TMB Solution to each well, cover wells with Adhesive Films (white), and incubate at room temperature for 15-20 minutes in the dark. If the room temperature is low, prolong the incubation time until the standard sample exhibits significant color change. If the concentration of target protein in sample is high, color change will occur soon.
   j. Add 50μl of Stop Solution to each well. Read the absorbance at 450nm immediately after mixing.

4. **Analysis.**
   a. Calculate the average A450 value for each standard and sample. Duplicates should be within 20 percent of the mean value.

b. Subtract the A450 value of blank control from the A450 values of standards and samples (This step can be omitted if there is no blank control).

c. Generate the standard curve by plotting the concentrations of standard on the abscissa and their corresponding A450 values on the ordinate (Figure 3).

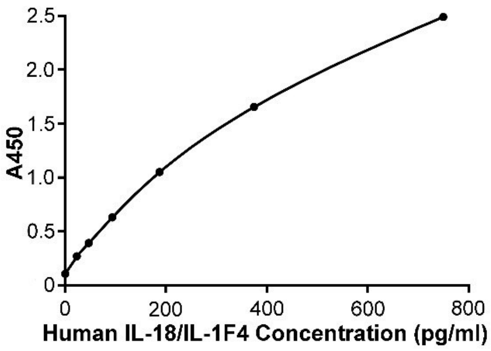


**Figure 3.** The standard curve of Beyotime's Human IL-18/IL-1F4 ELISA Kit (PI558). This figure is for reference only, which may vary due to different experimental conditions.

d. Determine the concentrations of human IL-18/IL-1F4 in samples from the standard curve based on their A450 values.

***Note:*** *Dilute samples producing signals greater than the upper limit of the standard curve in Standard Dilution Buffer and reanalyze. Multiply the concentration by the dilution factor.*

## Related Products:

| Cat. No. | Product Name | Pack Size |
|---|---|---|
| PI553 | Mouse IL-18 ELISA Kit | 96T |
| PI558 | Human IL-18/IL-1F4 ELISA Kit | 96T |

Version 2020.01.10

# Human OPG (Osteoprotegerin) ELISA Kit

Catalog No: E-EL-H1341
Size: 96T/48T/24T/96T*5

## Intended use

This ELISA kit applies to the in vitro quantitative determination of Human OPG concentrations in serum, plasma and other biological fluids.

## Character

| Item | |
|---|---|
| Sensitivity | 0.09 ng/mL |
| Detection Range | 0.16-10 ng/mL |
| Specificity | This kit recognizes Human OPG in samples. No significant cross-reactivity or interference between Human OPG and analogues was observed |
| Repeatability | Coefficient of variation is < 10% |

## Test principle

This ELISA kit uses the Sandwich-ELISA principle. The micro ELISA plate provided in this kit has been pre-coated with an antibody specific to Human OPG. Samples (or Standards) are added to the micro ELISA plate wells and combined with the specific antibody. Then a biotinylated detection antibody specific for Human OPG and Avidin-Horseradish Peroxidase (HRP) conjugate are added successively to each micro plate well and incubated. Free components are washed away. The substrate solution is added to each well. Only those wells that contain Human OPG, biotinylated detection antibody and Avidin-HRP conjugate will appear blue in color. The enzyme-substrate reaction is terminated by the addition of stop solution and the color turns yellow. The optical density (OD) is measured spectrophotometrically at a wavelength of 450±2 nm. The OD value is proportional to the concentration of Human OPG. You can calculate the concentration of Human OPG in the samples by comparing the OD of the samples to the standard curve.

## Kit components & Storage

An unopened kit can be stored at 2-8℃ for 12 months. After opening, store the items separately according to the following conditions.

| Item | Specifications | Storage |
|---|---|---|
| Micro ELISA Plate (Dismountable) | 96T: 8 wells ×12 strips<br>48T: 8 wells ×6 strips<br>24T: 8 wells ×3 strips<br>96T*5: 5 plates, 96T | -20℃, up to expiry date (12 months) |
| Reference Standard | 96T: 2 vials<br>48T/24T: 1 vial<br>96T*5: 10 vials | |
| Concentrated Biotinylated Detection Ab(100×) | 96T: 1 vial, 120 μL<br>48T/24T: 1 vial, 60 μL<br>96T*5: 5 vials, 120 μL | |
| Concentrated HRP Conjugate (100×) | 96T: 1 vial, 120 μL<br>48T/24T: 1 vial, 60 μL<br>96T*5: 5 vials, 120 μL | |
| Reference Standard & Sample Diluent | 96T/48T/24T: 1 vial, 20 mL<br>96T*5: 5 vials, 20 mL | 2-8℃, up to expiry date (12 months) |
| Biotinylated Detection Ab Diluent | 96T/48T/24T: 1 vial, 14 mL<br>96T*5: 5 vials, 14 mL | |
| HRP Conjugate Diluent | 96T/48T/24T: 1 vial, 14 mL<br>96T*5: 5 vials, 14 mL | |
| Concentrated Wash Buffer(25×) | 96T/48T/24T: 1 vial, 30 mL<br>96T*5: 5 vials, 30 mL | |
| Substrate Reagent | 96T/48T/24T: 1 vial, 10 mL<br>96T*5: 5 vials, 10 mL | |
| Stop Solution | 96T/48T/24T: 1 vial, 10 mL<br>96T*5: 5 vials, 10 mL | |
| Plate Sealer | 96T/48T/24T: 5 pieces<br>96T*5: 25 pieces | |
| Product Description | 1 copy | |
| Certificate of Analysis | 1 copy | |

**Note: Concentrated HRP Conjugate(100×)** and **Substrate Reagent** should be stored away from light.

All reagent bottle caps must be tightened to prevent evaporation and microbial pollution. The volume of reagents in partial shipments is a little more than the volume marked on the label, please use accurate measuring equipment instead of directly pouring into the vial(s).

## Other supplies required

Microplate reader with 450 nm wavelength filter
High-precision transfer pipette, EP tubes and disposable pipette tips
Incubator capable of maintaining 37℃
Deionized or distilled water
Absorbent paper
Loading slot

## Sample collection

**Serum:** Allow samples to clot for 1 hour at room temperature or overnight at 2-8℃before centrifugation for 20 min at 1000×g at 2-8℃. Collect the supernatant to carry out the assay.
**Plasma:** Collect plasma using EDTA-$Na_2$ as an anticoagulant. Centrifuge samples for 15 min at 1000×g at 2-8℃ within 30 min of collection. Collect the supernatant to carry out the assay.
**Tissue homogenates:** It is recommended to get detailed references from the literature before analyzing different tissue types. For general information, hemolyzed blood may affect the results, so the tissues should be minced into small pieces and rinsed in ice-cold PBS (0.01M, pH=7.4) to remove excess blood thoroughly. Tissue pieces should be weighed and then homogenized in PBS (tissue weight (g): PBS (mL) volume=1:9) with a glass homogenizer on ice. To further break down the cells, you can sonicate the suspension with an ultrasonic cell disrupter or subject it to freeze-thaw cycles. The homogenates are then centrifuged for 5-10 min at 5000×g at 2-8℃ to get the supernatant.
**Cell lysates:** For adherent cells, gently wash the cells with moderate amount of pre-cooled PBS and dissociate the cells using trypsin. Collect the cell suspension into a centrifuge tube and centrifuge for 5 min at 1000×g. Discard the medium and wash the cells 3 times with pre-cooled PBS. For each $1\times10^6$ cells, add 150-250 μL of pre-cooled PBS to keep the cells suspended. Repeat the freeze-thaw process several times or use an ultrasonic cell disrupter until the cells are fully lysed. Centrifuge for 10 min at 1500×g at 2-8℃. Remove the cell fragments, collect the supernatant to carry out the assay.
**Cell culture supernatant or other biological fluids:** Centrifuge samples for 20 min at 1000×g at 2-8℃. Collect the supernatant to carry out the assay.

**Recommended reagents for sample preparation:** PMSF Protease Inhibitor (Cat No. E-EL-SR002), 0.25% Trypsin Solution (Cat No. E-EL-SR001).

# Note

## ■ Note for kit

1) For research use only. Not for use in diagnostic procedures.
2) Please wear lab coats, eye protection and latex gloves for protection. Please perform the experiment following the national security protocols of biological laboratories, especially when detecting blood samples or other bodily fluids.
3) A freshly opened ELISA plate may appear a water-like substance, which is normal and will not have any impact on the experimental results. Return the unused wells to the foil pouch and store according to the conditions suggested in the above table.
4) Do not reuse the reconstituted standard, biotinylated detection Ab working solution, HRP conjugate working solution. The unspent undiluted concentrated biotinylated detection Ab (100×) and other stock solutions should be stored according to the storage conditions in the above table.
5) The microplate reader should be able to be installed with a filter that can detect the wave length at 450±2 nm. The optical density should be within 0-3.5. Follow the Instructions of the Microplate Reader for set-up and preheat it for 15 min before OD measurement.
6) **Do not mix or substitute reagents with those from other lots or sources.**
7) Change pipette tips in between adding of each standard level, between sample adding and between reagent adding. Also, use separate reservoirs for each reagent.
8) The kit should not be used beyond the expiration date on the kit label.

## ■ Note for sample

1) Tubes for blood collection should be disposable and be non-endotoxin. Samples with high hemolysis or much lipid are not suitable for ELISA assay.
2) Samples should be assayed within 7 days when stored at 2-8℃, otherwise samples must be divided up and stored at -20℃ (≤1 month) or -80℃ (≤3 months). Avoid repeated freeze-thaw cycles. Prior to assay, the frozen samples should be slowly thawed and centrifuged to remove precipitates.
3) Please predict the concentration before assaying. If the sample concentration is not within the range of the standard curve, users must determine the optimal sample dilutions for their particular experiments.
4) If the sample type is not included in the manual, a preliminary experiment is suggested to verify the validity.
5) If a lysis buffer is used to prepare tissue homogenates or cell lysates, there is a possibility of causing a deviation due to the introduced chemical substance.
6) Some recombinant protein may not be detected due to a mismatching with the coated antibody or detection antibody.

## Dilution method

Please predict the concentration range of samples in advance, and determine the dilution ratio through preliminary experiments or technical support recommendations.

If your test sample needs dilution, please refer to the dilution method as follows:
For 100 fold dilution: One-step dilution. Add 5 μL sample to 495 μL sample diluent to yield 100 fold dilution.
For 1000 fold dilution: Two-step dilution. Add 5 μL sample to 95 μL sample diluent to yield 20 fold dilution, then add 5 μL 20 fold diluted sample to 245 μL sample diluent, after this, the neat sample has been diluted at 1000 fold successfully.
For 100000 fold dilution: Three-step dilution. Add 5 μL sample to 195 μL sample diluent to yield 40 fold dilution, then add 5 μL 40 fold diluted sample to 245 μL sample diluent to yield 50 fold dilution, and finally add 5 μL 2000 fold diluted sample to 245 μL sample diluent, after this, the neat sample has been diluted at 100000 fold successfully.

## Reagent preparation

1. Bring all reagents to room temperature (18-25℃) before use. If the kit will not be used up in one assay, please only take out the necessary strips and reagents for present experiment, and store the remaining strips and reagents at required condition.
2. **Wash Buffer:** Dilute 30 mL of Concentrated Wash Buffer with 720 mL of deionized or distilled water to prepare 750 mL of Wash Buffer. Note: if crystals have formed in the concentrate, warm it in a 40℃ water bath and mix it gently until the crystals have completely dissolved. For same day use only.
3. **Standard working solution:** Centrifuge the standard at 10,000×g for 1 min. Add 1 mL of Reference Standard &Sample Diluent, let it stand for 10 min and invert it gently several times. After it dissolves fully, mix it thoroughly with a pipette. This reconstitution produces a working solution of 10 ng/mL (or add 1 mL of Reference Standard &Sample Diluent, let it stand for 1-2 min and then mix it thoroughly with a vortex meter of low speed. Bubbles generated during vortex could be removed by centrifuging at a relatively low speed). Then make serial dilutions as needed. The recommended dilution gradient is as follows: 10, 5, 2.5, 1.25, 0.63, 0.32, 0.16, 0 ng/mL. Dilution method: Take 7 EP tubes, add 500 μL of Reference Standard & Sample Diluent to each tube. Pipette 500 μL of the 10 ng/mL working solution to the first tube and mix up to produce a 5 ng/mL working solution. Pipette 500 μL of the solution from the former tube into the latter one according to this step. The illustration on the next page is for reference. Note: the last tube is regarded as a blank. Don't pipette solution into it from the former tube.

The working solution of the standard substance at 10 ng/mL after reconstitution should be aliquoted and stored at -20 ℃. It should be used up within half a month and repeated freeze-thaw should be avoided.

Gradient diluted standard working solution should be prepared just before use.

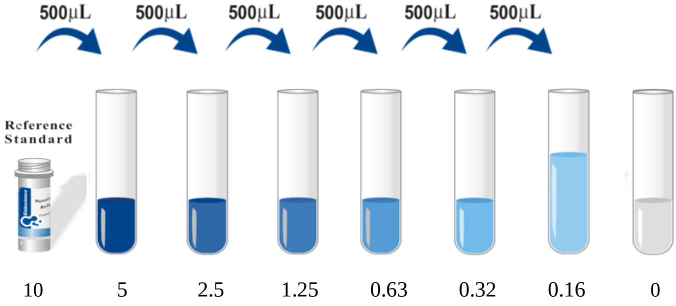


4. **Biotinylated Detection Ab working solution**: Calculate the required amount before the experiment (100 μL/well). In preparation, slightly more than calculated should be prepared. Centrifuge the Concentrated Biotinylated Detection Ab at 800×g for 1 min, then dilute the 100× Concentrated Biotinylated Detection Ab to 1× working solution with Biotinylated Detection Ab Diluent (Concentrated Biotinylated Detection Ab: Biotinylated Detection Ab Diluent= 1: 99). The working solution should be prepared just before use.
5. **HRP Conjugate working solution:** HRP Conjugate is HRP conjugated avidin. Calculate the required amount before the experiment (100 μL/well). In preparation, slightly more than calculated should be prepared. Centrifuge the Concentrated HRP Conjugate at 800×g for 1 min, then dilute the 100× Concentrated HRP Conjugate to 1× working solution with HRP Conjugate Diluent (Concentrated HRP Conjugate: HRP Conjugate Diluent= 1: 99). The working solution should be prepared just before use.

## Assay procedure

1. Determine wells for **diluted standard**, **blank** and **sample**. Add 100 μL each dilution of standard, blank and sample into the appropriate wells (It is recommended that all samples and standards be assayed in duplicate. It is recommended to determine the dilution ratio of samples through preliminary experiments or technical support recommendations). Cover the plate with the sealer provided in the kit. Incubate for 90 min at 37˚C. Note: solutions should be added to the bottom of the micro ELISA plate well, avoid touching the inside wall and causing foaming as much as possible.
2. Decant the liquid from each well, do not wash. Immediately add 100 μL of **Biotinylated Detection Ab working solution** to each well. Cover the plate with a new sealer. Incubate for 1 hour at 37 ℃.
3. Decant the solution from each well， add 350 μL of **wash buffer** to each well. Soak for 1 min and aspirate or decant the solution from each well and pat it dry against clean absorbent paper. Repeat this wash step 3 times. Note: a microplate washer can be used in this step and other wash steps. Make the tested strips in use immediately after the wash step. Do not allow wells to be dry.
4. Add 100 μL of **HRP Conjugate working solution** to each well. Cover the plate with a new sealer. Incubate for 30 min at 37 ℃.
5. Decant the solution from each well, repeat the wash process for 5 times as conducted in step 3.
6. Add 90 μL of **Substrate Reagent** to each well. Cover the plate with a new sealer. Incubate for about 15 min at 37 ℃. Protect the plate from light. Note: the reaction time can be shortened or extended according to the actual color change, but not more than 30 min. Preheat the Microplate Reader for about 15 min before OD measurement.
7. Add 50 μL of **Stop Solution** to each well. Note: adding the stop solution should be done in the same order as the substrate solution.
8. Determine the optical density (OD value) of each well at once with a micro-plate reader set to 450 nm.

# Assay Procedure Summary

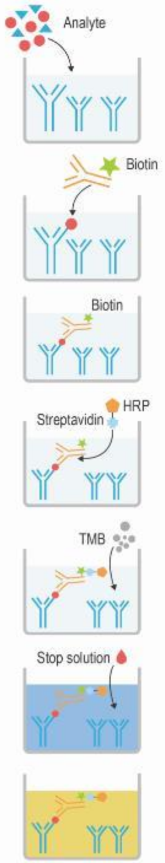


1. Add 100μL standard or sample to the wells. Incubate for 90 min at 37°C

2. Discard the liquid, immediately add 100μL Biotinylated Detection Ab working solution to each well. Incubate for 60 min at 37°C

3. Aspirate and wash the plate for 3 times

4. Add 100μL HRP conjugate working solution. Incubate for 30 min at 37°C. Aspirate and wash the plate for 5 times

5. Add 90μL Substrate Reagent. Incubate for 15 min at 37°C

6. Add 50μL Stop Solution

7. Read the plate at 450nm immediately. Calculation of the results

## Calculation of results

Average the duplicate readings for each standard and samples, then subtract the average zero standard optical density. Plot a four parameter logistic curve on log-log axis, with standard concentration on the x-axis and OD values on the y-axis.

If the OD of the sample surpasses the upper limit of the standard curve, you should re-test it with an appropriate dilution. The actual concentration is the calculated concentration multiplied by the dilution factor.

## Technical resources

More detailed ELISA experiment guidelines and routine problem analysis can be obtained through wechat QR code at the lower left.

If you have any technical problems, please feel free to contact our technical support (it is recommended to take pictures and save the experimental data in time. Keep the used plate and remaining reagents).

**Guidelines for ELISA**

**Wechat of technical support**

## Typical data

As the OD values of the standard curve may vary according to the conditions of the actual assay performance (e.g. operator, pipetting technique, washing technique or temperature effects), the operator should establish a standard curve for each test. Typical standard curve and data is provided below for reference only.

| ng/mL | OD | Corrected OD | Standard Curve |
|---|---|---|---|
| 10 | 2.302 | 2.226 | |
| 5 | 1.59 | 1.514 | |
| 2.5 | 0.938 | 0.862 | |
| 1.25 | 0.504 | 0.428 | |
| 0.63 | 0.311 | 0.235 | |
| 0.32 | 0.187 | 0.111 | |
| 0.16 | 0.133 | 0.057 | |
| 0 | 0.076 | - | |

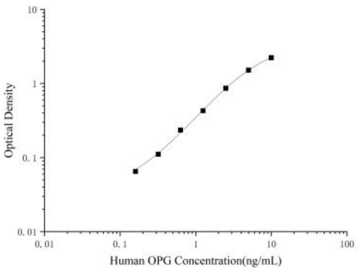


## Performance

- **Precision**

Intra-assay Precision (Precision within an assay): 3 samples with low, mid range and high level Human OPG were tested 20 times on one plate, respectively.

Inter-assay Precision (Precision between assays): 3 samples with low, mid range and high level Human OPG were tested on 3 different plates, 20 replicates in each plate, respectively.

| | Intra-assay Precision | | | Inter-assay Precision | | |
|---|---|---|---|---|---|---|
| Sample | 1 | 2 | 3 | 1 | 2 | 3 |
| n | 20 | 20 | 20 | 20 | 20 | 20 |
| Mean(ng/mL) | 0.55 | 1.01 | 4.75 | 0.55 | 1.1 | 4.42 |
| Standard deviation | 0.03 | 0.06 | 0.31 | 0.04 | 0.09 | 0.39 |
| CV (%) | 6.35 | 5.82 | 6.48 | 6.75 | 8.27 | 8.85 |

## ■ Recovery

The recovery of Human OPG spiked at three different levels in samples throughout the range of the assay was evaluated in various matrices.

| Sample Type | Range (%) | Average Recovery (%) |
|---|---|---|
| Serum (n=8) | 96-111 | 101 |
| EDTA plasma (n=8) | 96-110 | 102 |
| Cell culture media(n=8) | 86-101 | 93 |

## ■ Linearity

Samples were spiked with high concentrations of Human OPG and diluted with Reference Standard & Sample Diluent to produce samples with values within the range of the assay.

| | | Serum (n=5) | EDTA plasma (n=5) | Cell culture media(n=5) |
|---|---|---|---|---|
| 1:2 | Range (%) | 87-102 | 87-99 | 88-99 |
| | Average (%) | 94 | 92 | 94 |
| 1:4 | Range (%) | 97-115 | 82-94 | 96-114 |
| | Average (%) | 105 | 89 | 104 |
| 1:8 | Range (%) | 101-116 | 85-101 | 94-109 |
| | Average (%) | 106 | 92 | 100 |
| 1:16 | Range (%) | 96-110 | 82-91 | 94-109 |
| | Average (%) | 104 | 87 | 101 |

## Declaration

1. Limited by current conditions and scientific technology, we can't conduct comprehensive identification and analysis on all the raw material provided. So there might be some qualitative and technical risks for users using the kit.
2. This assay is designed to eliminate interference by factors present in biological samples. Until all factors have been tested in the ELISA immunoassay, the possibility of interference cannot be excluded.
3. The final experimental results will be closely related to the validity of products, operational skills of the operators, the experimental environments and so on. We are only responsible for the kit itself, but not for the samples consumed during the assay. The users should calculate the possible amount of the samples used in the whole test. Please reserve sufficient samples in advance.
4. To get the best results, please only use the reagents supplied by the manufacturer and strictly comply with the instructions.
5. Incorrect results may occur because of incorrect operations during the reagents preparation and loading, as well as incorrect parameter settings of the Micro-plate reader. Please read the instructions carefully and adjust the instrument prior to the experiment.
6. Even the same operator might get different results in two separate experiments. In order to get reproducible results, the operation of every step in the assay should be controlled.
7. Every kit has strictly passed QC test. However, results from end users might be inconsistent with our data due to some variables such as transportation conditions, different lab equipment, and so on. Intra-assay variance among kits from different batches might arise from the above reasons too.
8. Kits from different manufacturers or other methods for testing the same analyte could bring out inconsistent results, since we haven’t compared our products with those from other manufacturers.
9. The kit is designed for research use only, we will not be responsible for any issues if the kit is applied in clinical diagnosis or any other related procedures.

Version 3a Last updated 18 January 2024

# ab213758 – Human CD244 ELISA Kit (2B4)

For the quantitative detection of Human CD244 in cell culture supernatants, cell lysates, serum and plasma (heparin, EDTA).

This product is for research use only and is not intended for diagnostic use.

Copyright © 2024 Abcam. All rights reserved

# Table of Contents


1. Overview 1
2. Protocol Summary 2
3. Precautions 3
4. Storage and Stability 3
5. Limitations 4
6. Materials Supplied 4
7. Materials Required, Not Supplied 5
8. Technical Hints 6
9. Reagent Preparation 7
10. Standard Preparation 9
11. Sample Preparation 10
12. Assay Procedure 11
13. Calculations 13
14. Typical data 14
15. Typical sample values 15
16. Troubleshooting 16
17. Notes 17


Copyright © 2024 Abcam. All rights reserved

# 1. Overview

The Human CD244 Enzyme-Linked Immunosorbent Assay (ELISA) kit (2B4) (ab213758) is designed for the quantitative measurement of Human CD244 in cell culture supernatants, cell lysates, serum and plasma (heparin, EDTA).

The ELISA kit is based on standard sandwich enzyme-linked immunosorbent assay technology. A monoclonal antibody from mouse specific for CD244/2B4 has been pre-coated onto 96-well plates. Standards (Expression system for standard: NSO; Immunogen sequence: C22-R221) and test samples are added to the wells, a biotinylated detection polyclonal antibody from goat specific for CD244/2B4 is added subsequently and then followed by washing with 1X Wash Buffer. Avidin-Biotin-Peroxidase Complex is added and unbound conjugates are washed away with 1X Wash Buffer. HRP substrate TMB is used to visualize HRP enzymatic reaction. TMB is catalyzed by HRP to produce a blue color product that changed into yellow after adding acidic TMB Stop Solution. The density of yellow is proportional to the Human CD244/2B4 amount of sample captured in plate.

CD244 (Cluster of Differentiation 244) is a human protein encoded by the CD244 gene. It is also known as Natural Killer Cell Receptor 2B4. Members of the CD2 family adhere to each other instead, the cell surface glycoprotein 2B4 is related to CD2 and is implicated in the regulation of NK and T-cell function. The 2B4 gene was mapped to 1q22 on gene map, where SLAM, CD48, CD84 and LY9 are also located. Using recombinant human NK cell-activating ligand 2B4 fused to domains 3 and 4 of rodent Cd4 and flow cytometric analysis, It was found that CD48 binds to 2B4. BIAcore surface plasmon resonance analysis showed that the affinity of CD48 for 2B4 is approximately 10-fold higher than that shown for CD48 and CD2.

## 2. Protocol Summary

Prepare all reagents, samples, and standards as instructed

⇩

Add 100 µL standard or sample to appropriate wells

Incubate at 37°C for 90 minutes

⇩

Discard plate content. Do not wash.

Add 100 µL biotinylated Antibody in to all wells

Incubate at 37°C for 60 minutes

⇩

Wash each well three times with 300 µL of 1X Wash Buffer

⇩

Add 100 µL ABC working solution

Incubate at 37°C for 30 minutes

⇩

Wash each well five times with 300 µL of 1X Wash Buffer

Add 90 µL of prepared TMB Color Developing Agent

Incubate at 37°C in dark for 25-30 minutes

⇩

Add 100 µL TMB Stop Solution and read OD at 450 nm within 30 minutes

## 3. Precautions

**Please read these instructions carefully prior to beginning the ELISA assay.**

- All kit components have been formulated and quality control tested to function successfully as a kit.
- We understand that, occasionally, experimental protocols might need to be modified to meet unique experimental circumstances. However, we cannot guarantee the performance of the product outside the conditions detailed in this protocol booklet.
- Reagents should be treated as possible mutagens and should be handled with care and disposed of properly. Please review the Safety Datasheet (SDS) provided with the product for information on the specific components.
- Observe good laboratory practices. Gloves, lab coat, and protective eyewear should always be worn. Never pipette by mouth. Do not eat, drink or smoke in the laboratory areas.
- All biological materials should be treated as potentially hazardous and handled as such. They should be disposed of in accordance with established safety procedures.

## 4. Storage and Stability

**Store ELISA kit at -20°C immediately upon receipt.**

Refer to list of materials supplied for storage conditions of individual components. Observe the storage conditions for individual prepared components in the Materials Supplied section.

Aliquot components in working volumes before storing at the recommended temperature.

## 5. Limitations

- ELISA kit intended for research use only. Not for use in diagnostic procedures.
- Do not mix or substitute reagents or materials from other kit lots or vendors. Kits are QC tested as a set of components and performance cannot be guaranteed if utilized separately or substituted.

## 6. Materials Supplied

| Item | Quantity | Storage Condition (Before prep) | Storage Condition (After prep) |
|---|---|---|---|
| Anti-Human CD244 coated Microplate (12 x 8 wells) | 1 x 96 well plate | -20°C | -20°C |
| Lyophilized recombinant Human CD244 standard | 2 x 1 vial | -20°C | -20°C |
| Biotinylated anti- Human CD244 antibody | 100 µL | -20°C | -20°C |
| Avidin-Biotin-Peroxidase Complex (ABC) | 100 µL | -20°C | -20°C |
| Sample diluent buffer | 30 mL | -20°C | -20°C |
| Antibody diluent buffer | 12 mL | -20°C | -20°C |
| ABC diluent buffer | 12 mL | -20°C | -20°C |
| TMB Color Developing Agent | 10 mL | -20°C | -20°C |
| TMB Stop Solution | 10 mL | -20°C | -20°C |
| Adhesive Plate Seal | 4 | -20°C | -20°C |
| Wash Buffer (25X) | 20 mL | -20°C | -20°C |

## 7. Materials Required, Not Supplied

These materials are not included in the kit, but will be required to successfully perform this assay:

- Microplate reader capable of measuring absorbance at 450 nm.
- Automated plate washer.
- Multi- and single-channel pipettes.
- Clean tubes and Eppendorf tubes.

## 8. Technical Hints

- Samples generating values higher than the highest standard should be further diluted in the appropriate sample dilution buffers.
- Avoid foaming or bubbles when mixing or reconstituting components.
- Avoid cross contamination of samples or reagents by changing tips between sample, standard and reagent additions.
- Ensure plates are properly sealed or covered during incubation steps.
- Don't let the 96-well plate dry, for a dry plate will inactivate active components on plate.
- Complete removal of all solutions and buffers during wash steps is necessary to minimize background.
- All samples should be mixed thoroughly and gently.
- Avoid multiple freeze/thaw of samples.
- When generating positive control samples, it is advisable to change pipette tips after each step.
- Before using the kit, spin tubes and bring down all components to the bottom of tubes.
- In order to avoid marginal effect of plate incubation due to temperature difference (reaction may be stronger in the marginal wells), it is suggested that the diluted ABC and TMB solution will be pre-warmed in 37°C for 30 minutes before using.
- **To avoid high background always add samples or standards to the well before the addition of the antibody cocktail.**
- **This kit is sold based on number of tests. A 'test' simply refers to a single assay well. The number of wells that contain sample, control or standard will vary by product. Review the protocol completely to confirm this kit meets your requirements. Please contact our Technical Support staff with any questions.**

## 9. Reagent Preparation

- **Equilibrate all reagents to room temperature (18-25°C) prior to use. The kit contains enough reagents for 96 wells.**
- **Prepare only as much reagent as is needed on the day of the experiment.**

**9.1 Anti-human CD244 coated Microplate (12 x 8 wells)**
One plate of 96 wells. Ready to use. Store at -20°C.

**9.2 Lyophilized recombinant Human CD244 standard (2 x 10 ng)**

9.2.1 CD244 standard solution should be prepared no more than 2 hours prior to the experiment. Two tubes of CD244 standard (2 x 10 ng) are included in each kit. Use one tube for each experiment.

9.2.2 Add 1 mL sample diluent buffer into one tube to create 10,000 ng/mL of Human CD244 stock solution. Keep the tube at room temperature for 10 minutes and mix thoroughly.

**9.3 Biotinylated anti- Human CD244 antibody**
The solution should be prepared no more than 2 hours prior to the experiment.

9.3.1 The total volume should be: 100 µL/well x (the number of wells). (Allowing 100 µL – 200 µL more than total volume)

9.3.2 Biotinylated anti-Human CD244 antibody should be diluted in 1:100 with the antibody diluent buffer and mixed thoroughly. (i.e. Add 1 µL Biotinylated Anti-Human CD244 antibody to 99 µL antibody diluent buffer.)

**9.4 Avidin-Biotin-Peroxidase Complex (ABC)**
Before use, briefly centrifuge the tubes in case any of the contents are trapped in the lid or sticking to the tube walls. The solution should be prepared no more than 1 hour prior to the experiment.

9.4.1 The total volume should be: 100 µL/well x (the number of wells). (Allowing 100 µL - 200 µL more than total volume)

9.4.2 Avidin- Biotin-Peroxidase Complex (ABC) should be diluted in 1:100 with the ABC dilution buffer and mixed thoroughly. (i.e. Add 1 µL ABC to 99 µL ABC diluent buffer.)

## 9.5 Sample diluent buffer

30 mL. Ready to use. Store at -20°C.

## 9.6 Antibody diluent buffer

12 mL. Ready to use. Store at -20°C.

## 9.7 ABC diluent buffer

12 mL. Ready to use. Store at -20°C.

## 9.8 TMB Color Developing Agent

10 mL. Ready to use. Store at -20°C.

## 9.9 TMB Stop Solution

10 mL. Ready to use. Store at -20°C.

## 9.10 1X Wash Buffer

Prepare 500 mL of working 1X Wash Buffer by diluting 20 ml of the supplied Wash Buffer (25X) with 480 ml of deionized or distilled water. If crystals have formed in the concentrate, warm to room temperature and mix it gently until crystals have completely dissolved.

# 10. Standard Preparation

**10.1** Prepare a 1,000 pg/mL CD244 solution by adding 100 µL of the above 10,000 pg/mL CD244 stock solution into a tube with 900 µL sample diluent buffer and mix thoroughly.

**10.2** To prepare standards, label 6 Eppendorf tubes with 500 pg/mL, 250 pg/mL, 125 pg/mL, 62.5 pg/mL, 31.25 pg/mL and 15.63 pg/mL respectively. The sample diluent serves as a zero standard.

**10.3** Aliquot 300 µL of the sample diluent buffer into each tube.

**10.4** Add 300 µL of the above 1000 pg/mL CD244 solution into 2nd tube and mix.

**10.5** Transfer 300 µL from 2nd tube to 3rd tube and mix. Transfer 300 µL from 3rd tube to 4th tube and mix, and so on.

| Tube # | Volume to dilute | Volume of diluent | Concentratio n (pg/mL) |
|---|---|---|---|
| 1 | 100 µL of 10,000 pg/mL stock solution | 900 µL | 1000 |
| 2 | 300 µL of 1,000 pg/mL stock solution | 300 µL | 500 |
| 3 | 300 µL of tube #2 | 300 µL | 250 |
| 4 | 300 µL of tube #3 | 300 µL | 125 |
| 5 | 300 µL of tube #4 | 300 µL | 62.5 |
| 6 | 300 µL of tube #5 | 300 µL | 31.25 |
| 7 | 300 µL of tube #6 | 300 µL | 15.63 |
| 8 | Zero Standard | 600 µL | 0 |

Δ **Note:** The standard solutions are best used within 2 hours. The 10,000 pg/mL standard solution should be stored at 4°C for up to 12 hours, or at -20°C for up to 48 hours. Avoid repeated freeze-thaw cycles.

## 11. Sample Preparation

Store samples to be assayed within 24 hours at 4°C. For long-term storage, aliquot and freeze samples at -20°C. Avoid repeated freeze-thaw cycles.

- Cell lysates: After sufficient splitting, there should be no obvious cell sediment. Centrifuge cell lysates at approximately 10,000 x g for 5 minutes. Collect the cell lysate supernatants to go ahead.
- Serum: Allow the serum to clot in a serum separator tube (about 4 hours) at room temperature. Centrifuge at approximately 1,000 x g for 15 minutes. Analyze the serum immediately or aliquot and store samples at -20°C.
- Cell culture supernatant: Remove particulates by centrifugation, assay immediately or aliquot and store samples at -20°C.
- Plasma: Collect plasma using heparin or EDTA as an anticoagulant. Centrifuge for 15 minutes at 1,500 x g within 30 minutes of collection. Assay immediately or aliquot and store samples at -20°C.

It is recommended to estimate the concentration of the target protein in the sample and select a proper dilution factor so that the diluted target protein concentration falls near the middle of the linear regime in the standard curve. Dilute the sample using the provided diluent buffer. The following is a guideline for sample dilution. Several trials may be necessary in practice. The sample must be well mixed with the diluents buffer.

## 12. Assay Procedure

- **It is recommended to assay all standards, controls and samples in duplicate.**
- **The ABC working solution and TMB color developing agent must be kept warm at 37°C for 30 minutes before use. When diluting samples and reagents, they must be mixed completely and evenly. Standard CD244 detection curve should be prepared for each experiment. The user will decide sample dilution fold by crude estimation of CD244 amount in samples.**

**12.1** Aliquot 100 µL per well of the 1,000 pg/mL, 500 pg/mL, 250 pg/mL, 125 pg/mL, 62.50 pg/mL, 31.25 pg/mL, 15.63 pg/mL Human CD244 standard solutions into the pre-coated 96-well plate.

**12.2** Add 100 µL of the sample diluent buffer into the control well (Zero well).

**12.3** Add 100 µL of each properly diluted sample of Human cell culture supernatants, cell lysates, serum or plasma (heparin, EDTA) to each empty well. See "Sample Preparation" above for details. It is recommended that each Human CD244 standard solution and each sample be measured in duplicate.

**12.4** Seal the plate with a new adhesive cover provided and incubate at 37°C for 90 minutes.

**12.5** Remove the cover, discard plate content, and blot the plate onto paper towels or other absorbent material. Do NOT let the wells completely dry at any time.

**12.6** Add 100 µL of biotinylated anti-Human CD244 antibody working solution into each well, seal the plate with a new adhesive cover provided and incubate at 37°C for 60 minutes.

**12.7** Wash plate 3 times with 1X Wash Buffer, and each time let washing buffer stay in the wells for 1 minute. Discard the washing buffer and blot the plate onto paper towels or other absorbent material. (Plate Washing Method: Discard the solution in the plate without touching the side walls. Blot the plate onto paper towels or other absorbent material. Soak each well with at least 300 µL of 1X Wash Buffer for 1~2 minutes. Repeat this process two additional times for a total of three washes.

**Note:** For automated washing, aspirate all wells and wash three times with 1X Wash Buffer, overfilling wells with 1X Wash

Buffer. Blot the plate onto paper towels or other absorbent material.)

**12.8** Add 100 µL of prepared ABC working solution into each well, seal the plate with a new adhesive cover provided and incubate at 37°C for 30 minutes.

**12.9** Wash plate 5 times with 1X Wash Buffer, and each time let washing buffer stay in the wells for 1-2 minutes. Discard the washing buffer and blot the plate onto paper towels or other absorbent material. (See Step 12.7 for plate washing method.)

**12.10** Add 90 µL of prepared TMB color developing agent into each well, seal the plate with a new adhesive cover and incubate at 37°C in dark for 25-30 minutes.

Δ **Note:** For reference only, the optimal incubation time should be determined by end user. And the shades of blue can be seen in the wells with the four most concentrated Human CD244 standard solutions; the other wells show no obvious color.

**12.11** Add 100 µL of prepared TMB Stop Solution into each well. The color changes into yellow immediately.

**12.12** Read the O.D. absorbance at 450 nm in a microplate reader within 30 minutes after adding the TMB Stop Solution.

# 13. Calculations

The standard curve can be plotted as the relative O.D.450 of each standard solution (Y) vs. the respective concentration of the standard solution (X). The Human CD244 concentration of the samples can be interpolated from the standard curve.

(the relative O.D.450) = (the O.D.450 of each well) – (the O.D.450 of Zero well).

Δ **Note:** if the samples measured were diluted, multiply the dilution factor to the concentrations from interpolation to obtain the concentration before dilution.

# 14. Typical data

**Typical standard** curve – Data provided for demonstration purposes only. A new standard curve must be generated for each assay performed.

| Sample | Human CD244 (pg/mL) | O.D. |
|---|---|---|
| 1 | 0 | 0.025 |
| 2 | 15.63 | 0.199 |
| 3 | 31.25 | 0.292 |
| 4 | 62.50 | 0.464 |
| 5 | 125 | 0.934 |
| 6 | 250 | 1.510 |
| 7 | 500 | 2.026 |
| 8 | 1,000 | 2.547 |

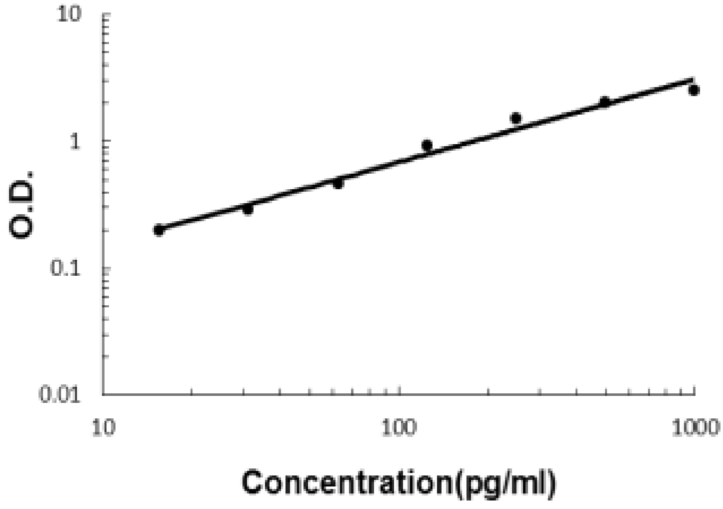


**Figure 1**. Human CD244 ELISA Kit (2B4) (ab213758) Standard Curve.

# 15. Typical sample values

## Sensitivity –

The biological sensitivity of the assay is <10 pg/mL.

The range is 15.625 pg/mL – 1,000 pg/mL.

## Precision –

**Intra-assay precision:** (Precision within an assay) Three samples of known concentration were tested on one plate to assess intra-assay precision.

| Sample | Number of measures | Mean (ng/mL) | Standard Deviation | CV% |
|---|---|---|---|---|
| 1 | 16 | 0.45 | 0.022 | 4.9 |
| 2 | 16 | 1.2 | 0.076 | 6.3 |
| 3 | 16 | 3.2 | 0.186 | 5.8 |

**Inter-assay precision:** (Precision between assays) Three samples of known concentration were tested in separate assays to assess inter-assay precision.

| Sample | Number of assays | Mean (ng/mL) | Standard Deviation | CV% |
|---|---|---|---|---|
| 1 | 24 | 0.43 | 0.027 | 6.2 |
| 2 | 24 | 1.3 | 0.098 | 7.5 |
| 3 | 24 | 2.9 | 0.194 | 6.7 |

## Specificity:

Natural and recombinant Human CD244.

## Cross-reactivity:

There is no detectable cross-reactivity with other relevant proteins.

| Problem | Cause | Solution |
|---|---|---|
| Poor standard curve | Inaccurate Pipetting | Check Pipettes |
| | Improper standard dilution | Prior to opening, briefly spin the stock standard tube and dissolve the powder thoroughly by gentle mixing |
| Low Signal | Incubation times too brief | Ensure sufficient incubation times standard/sample incubation |
| | Inadequate reagent volumes or improper dilution | Check Pipettes and ensure correct preparation |
| | Incubation times with TMB Color Developing Agent too brief | Ensure sufficient incubation time until blue color develops prior addition of TMB Stop Solution |
| Large CV | Plate is insufficiently washed | Review manual for proper wash technique. If using a plate washer, check all ports for obstructions. |
| | Contaminated wash buffer | Prepare fresh wash buffer |
| Low sensitivity | Improper storage of the ELISA kit | All components 4°C. Keep TMB substrate solution protected from light. |

# 16. Troubleshooting

# 17. Notes

## Technical Support

Copyright © 2024 Abcam. All Rights Reserved. The Abcam logo is a registered trademark. All information / detail is correct at time of going to print.

For all technical or commercial enquiries please go to:
www.abcam.com/contactus
www.abcam.cn/contactus (China)
www.abcam.co.jp/contactus (Japan)

Copyright © 2024 Abcam. All rights reserved

# fT4 (Free Thyroxine) ELISA Kit

Catalog No: E-EL-0122

Size: 96T/48T/24T/96T*5

## Intended use

This ELISA kit applies to the in vitro quantitative determination of fT4 concentrations in serum, plasma and other biological fluids.

## Character

| Item | |
|---|---|
| Sensitivity | 0.94 pg/mL |
| Detection Range | 1.56-100 pg/mL |
| Specificity | This kit recognizes fT4 in samples. No significant cross-reactivity or interference between fT4 and analogues was observed |
| Repeatability | Coefficient of variation is < 10% |

## Test principle

This ELISA kit uses the Competitive-ELISA principle. The micro ELISA plate provided in this kit has been pre-coated with fT4. During the reaction, fT4 in samples or Standard competes with a fixed amount of fT4 on the solid phase supporter for sites on the Biotinylated Detection Ab specific to fT4. Excess conjugate and unbound sample or standard are washed from the plate, and Avidin conjugated to Horseradish Peroxidase (HRP) are added to each microplate well and incubated. Then a TMB substrate solution is added to each well. The enzyme-substrate reaction is terminated by the addition of stop solution and the color change is measured spectrophotometrically at a wavelength of 450±2 nm. The concentration of fT4 in the samples is then determined by comparing the OD of the samples to the standard curve.

## Kit components & Storage

An unopened kit can be stored at 2-8℃ for 12 months. After opening, store the items separately according to the following conditions.

| Item | Specifications | Storage |
|---|---|---|
| Micro ELISA Plate (Dismountable) | 96T: 8 wells ×12 strips<br>48T: 8 wells ×6 strips<br>24T: 8 wells ×3 strips<br>96T*5: 5 plates, 96T | -20℃, up to expiry date (12 months) |
| Reference Standard | 96T: 2 vials<br>48T/24T: 1 vial<br>96T*5: 10 vials | |
| Concentrated Biotinylated Detection Ab(100×) | 96T: 1 vial, 120 μL<br>48T/24T: 1 vial, 60 μL<br>96T*5: 5 vials, 120 μL | |
| Concentrated HRP Conjugate (100×) | 96T: 1 vial, 120 μL<br>48T/24T: 1 vial, 60 μL<br>96T*5: 5 vials, 120 μL | |
| Reference Standard & Sample Diluent | 96T/48T/24T: 1 vial, 20 mL<br>96T*5: 5 vials, 20 mL | 2-8℃, up to expiry date (12 months) |
| Biotinylated Detection Ab Diluent | 96T/48T/24T: 1 vial, 14 mL<br>96T*5: 5 vials, 14 mL | |
| HRP Conjugate Diluent | 96T/48T/24T: 1 vial, 14 mL<br>96T*5: 5 vials, 14 mL | |
| Concentrated Wash Buffer(25×) | 96T/48T/24T: 1 vial, 30 mL<br>96T*5: 5 vials, 30 mL | |
| Substrate Reagent | 96T/48T/24T: 1 vial, 10 mL<br>96T*5: 5 vials, 10 mL | |
| Stop Solution | 96T/48T/24T: 1 vial, 10 mL<br>96T*5: 5 vials, 10 mL | |
| Plate Sealer | 96T/48T/24T: 5 pieces<br>96T*5: 25 pieces | |
| Product Description | 1 copy | |
| Certificate of Analysis | 1 copy | |

**Note: Concentrated HRP Conjugate(100×)** and **Substrate Reagent** should be stored away from light.

All reagent bottle caps must be tightened to prevent evaporation and microbial pollution. The volume of reagents in partial shipments is a little more than the volume marked on the label, please use accurate measuring equipment instead of directly pouring into the vial(s).

## Other supplies required

Microplate reader with 450nm wavelength filter
High-precision transfer pipette, EP tubes and disposable pipette tips
Incubator capable of maintaining 37℃
Deionized or distilled water
Absorbent paper
Loading slot

## Sample collection

**Serum:** Allow samples to clot for 1 hour at room temperature or overnight at 2-8℃ before centrifugation for 20 min at 1000×g at 2-8℃. Collect the supernatant to carry out the assay.
**Plasma:** Collect plasma using EDTA-$Na_2$ as an anticoagulant. Centrifuge samples for 15 min at 1000×g at 2-8℃ within 30 min of collection. Collect the supernatant to carry out the assay.
**Tissue homogenates:** It is recommended to get detailed references from the literature before analyzing different tissue types. For general information, hemolyzed blood may affect the results, so the tissues should be minced into small pieces and rinsed in ice-cold PBS (0.01M, pH=7.4) to remove excess blood thoroughly. Tissue pieces should be weighed and then homogenized in PBS (tissue weight (g): PBS (mL) volume=1:9) with a glass homogenizer on ice. To further break down the cells, you can sonicate the suspension with an ultrasonic cell disrupter or subject it to freeze-thaw cycles. The homogenates are then centrifuged for 5-10 min at 5000×g at 2-8℃ to get the supernatant.
**Cell lysates:** For adherent cells, gently wash the cells with moderate amount of pre-cooled PBS and dissociate the cells using trypsin. Collect the cell suspension into a centrifuge tube and centrifuge for 5 min at 1000×g. Discard the medium and wash the cells 3 times with pre-cooled PBS. For each $1\times10^6$ cells, add 150-250 μL of pre-cooled PBS to keep the cells suspended. Repeat the freeze-thaw process several times or use an ultrasonic cell disrupter until the cells are fully lysed. Centrifuge for 10 min at 1500×g at 2-8℃. Remove the cell fragments, collect the supernatant to carry out the assay.
**Cell culture supernatant or other biological fluids:** Centrifuge samples for 20 min at 1000×g at 2-8℃. Collect the supernatant to carry out the assay.

**Recommended reagents for sample preparation:** PMSF Protease Inhibitor (Cat No. E-EL-SR002), 0.25% Trypsin Solution (Cat No. E-EL-SR001).

# Note

- **Note for kit**

1) For research use only. Not for use in diagnostic procedures.
2) Please wear lab coats, eye protection and latex gloves for protection. Please perform the experiment following the national security protocols of biological laboratories, especially when detecting blood samples or other bodily fluids.
3) A freshly opened ELISA plate may appear a water-like substance, which is normal and will not have any impact on the experimental results. Return the unused wells to the foil pouch and store according to the conditions suggested in the above table.
4) Do not reuse the reconstituted standard, biotinylated detection Ab working solution, HRP conjugate working solution. The unspent undiluted concentrated biotinylated detection Ab (100×) and other stock solutions should be stored according to the storage conditions in the above table.
5) The microplate reader should be able to be installed with a filter that can detect the wave length at 450±2 nm. The optical density should be within 0-3.5. Follow the Instructions of the Microplate Reader for set-up and preheat it for 15 min before OD measurement.
6) **Do not mix or substitute reagents with those from other lots or sources.**
7) Change pipette tips in between adding of each standard level, between sample adding and between reagent adding. Also, use separate reservoirs for each reagent.
8) The kit should not be used beyond the expiration date on the kit label.

- **Note for sample**

1) Tubes for blood collection should be disposable and be non-endotoxin. Samples with high hemolysis or much lipid are not suitable for ELISA assay.
2) Samples should be assayed within 7 days when stored at 2-8℃, otherwise samples must be divided up and stored at -20℃ (≤1 month) or -80℃ (≤3 months). Avoid repeated freeze-thaw cycles. Prior to assay, the frozen samples should be slowly thawed and centrifuged to remove precipitates.
3) Please predict the concentration before assaying. If the sample concentration is not within the range of the standard curve, users must determine the optimal sample dilutions for their particular experiments.
4) If the sample type is not included in the manual, a preliminary experiment is suggested to verify the validity.
5) If a lysis buffer is used to prepare tissue homogenates or cell lysates, there is a possibility of causing a deviation due to the introduced chemical substance.
6) Some recombinant protein may not be detected due to a mismatching with the coated antibody or detection antibody.

## Dilution method

Please predict the concentration range of samples in advance, and determine the dilution ratio through preliminary experiments or technical support recommendations.

If your test sample needs dilution, please refer to the dilution method as follows:
For 100 fold dilution: One-step dilution. Add 5 μL sample to 495 μL sample diluent to yield 100 fold dilution.
For 1000 fold dilution: Two-step dilution. Add 5 μL sample to 95 μL sample diluent to yield 20 fold dilution, then add 5 μL 20 fold diluted sample to 245 μL sample diluent, after this, the neat sample has been diluted at 1000 fold successfully.
For 100000 fold dilution: Three-step dilution. Add 5 μL sample to 195 μL sample diluent to yield 40 fold dilution, then add 5 μL 40 fold diluted sample to 245 μL sample diluent to yield 50 fold dilution, and finally add 5 μL 2000 fold diluted sample to 245 μL sample diluent, after this, the neat sample has been diluted at 100000 fold successfully.

## Reagent preparation

1. Bring all reagents to room temperature (18-25℃) before use. If the kit will not be used up in one assay, please only take out the necessary strips and reagents for present experiment, and store the remaining strips and reagents at required condition.
2. **Wash Buffer:** Dilute 30 mL of Concentrated Wash Buffer with 720 mL of deionized or distilled water to prepare 750 mL of Wash Buffer. Note: if crystals have formed in the concentrate, warm it in a 40℃ water bath and mix it gently until the crystals have completely dissolved.
3. **Standard working solution:** Centrifuge the standard at 10,000×g for 1 min. Add 1 mL of Reference Standard &Sample Diluent, let it stand for 10 min and invert it gently several times. After it dissolves fully, mix it thoroughly with a pipette. This reconstitution produces a working solution of 100 pg/mL (or add 1 mL of Reference Standard &Sample Diluent, let it stand for 1-2 min and then mix it thoroughly with a vortex meter of low speed. Bubbles generated during vortex could be removed by centrifuging at a relatively low speed). Then make serial dilutions as needed. The recommended dilution gradient is as follows: 100、50、25、12.5、6.25、3.13、1.56、0 pg/mL.

   Dilution method: Take 7 EP tubes, add 500 μL of Reference Standard & Sample Diluent to each tube. Pipette 500 μL of the 100 pg/mL working solution to the first tube and mix up to produce a 50 pg/mL working solution. Pipette 500 μL of the solution from the former tube into the latter one according to this step. The illustration on the next page is for reference. Note: the last tube is regarded as a blank. Don't pipette solution into it from the former tube.

The working solution of the standard substance at 100 pg/mL after reconstitution should be aliquoted and stored at -20 ℃. It should be used up within half a month and repeated freeze-thaw should be avoided.

Gradient diluted standard working solution should be prepared just before use.

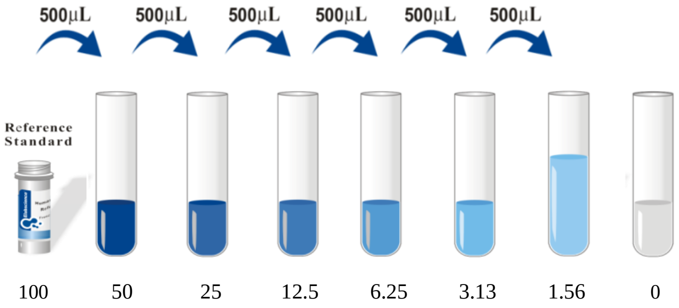


4. **Biotinylated Detection Ab working solution**: Calculate the required amount before the experiment (50 μL/well). In preparation, slightly more than calculated should be prepared. Centrifuge the Concentrated Biotinylated Detection Ab at 800×g for 1 min, then dilute the 100× Concentrated Biotinylated Detection Ab to 1× working solution with Biotinylated Detection Ab Diluent (Concentrated Biotinylated Detection Ab: Biotinylated Detection Ab Diluent= 1: 99). The working solution should be prepared just before use.
5. **HRP Conjugate working solution:** HRP Conjugate is HRP conjugated avidin. Calculate the required amount before the experiment (100 μL/well). In preparation, slightly more than calculated should be prepared. Centrifuge the Concentrated HRP Conjugate at 800×g for 1 min, then dilute the 100× Concentrated HRP Conjugate to 1× working solution with HRP Conjugate Diluent (Concentrated HRP Conjugate: HRP Conjugate Diluent= 1: 99). The working solution should be prepared just before use.

## Assay procedure

1. Determine wells for **diluted standard**, **blank** and **sample**. Add 50 μL each dilution of standard, blank and sample into the appropriate wells (It is recommended that all samples and standards be assayed in duplicate. It is recommended to determine the dilution ratio of samples through preliminary experiments or technical support recommendations). Immediately add 50 μL of **Biotinylated Detection Ab working solution** to each well. Cover the plate with the sealer provided in the kit. Incubate for 45 min at 37℃. Note: solutions should be added to the bottom of the micro ELISA plate well, avoid touching the inside wall and causing foaming as much as possible.
2. Decant the solution from each well， add 350 μL of **wash buffer** to each well. Soak for 1 min and aspirate or decant the solution from each well and pat it dry against clean absorbent paper. Repeat this wash step 3 times. Note: a microplate washer can be used in this step and other wash steps. Make the tested strips in use immediately after the wash step. Do not allow wells to be dry.
3. Add 100μL of **HRP Conjugate working solution** to each well. Cover the plate with a new sealer. Incubate for 30 min at 37 ℃.
4. Decant the solution from each well, repeat the wash process for 5 times as conducted in step 2.
5. Add 90 μL of **Substrate Reagent** to each well. Cover the plate with a new sealer. Incubate for about 15 min at 37 ℃. Protect the plate from light. Note: the reaction time can be shortened or extended according to the actual color change, but not more than 30 min. Preheat the Microplate Reader for about 15 min before OD measurement.
6. Add 50 μL of **Stop Solution** to each well. Note: adding the stop solution should be done in the same order as the substrate solution.
7. Determine the optical density (OD value) of each well at once with a micro-plate reader set to 450 nm.

# Assay Procedure Summary

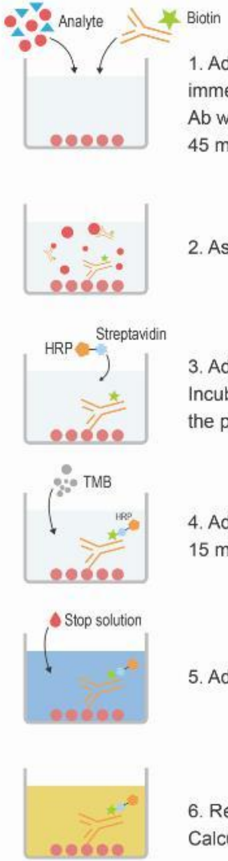


1. Add 50μL standard or sample to the wells, immediately add 50μL Biotinylated Detection Ab working solution to each well. Incubate for 45 min at 37°C

2. Aspirate and wash the plate for 3 times

3. Add 100μL HRP conjugate working solution. Incubate for 30 min at 37°C. Aspirate and wash the plate for 5 times

4. Add 90μL Substrate Reagent. Incubate for 15 min at 37°C

5. Add 50μL Stop Solution

6. Read the plate at 450nm immediately. Calculation of the results

## Calculation of results

Average the duplicate readings for each standard and samples. Plot a four parameter logistic curve on log-log axis, with standard concentration on the x-axis and OD values on the y-axis.

If the OD of the sample under the lowest limit of the standard curve, you should re-test it with an appropriate dilution. The actual concentration is the calculated concentration multiplied by the dilution factor.

## Technical resources

More detailed ELISA experiment guidelines and routine problem analysis can be obtained through wechat QR code at the lower left.

If you have any technical problems, please feel free to contact our technical support (it is recommended to take pictures and save the experimental data in time. Keep the used plate and remaining reagents).

**Guidelines for ELISA**

**Wechat of technical support**

## Typical data

As the OD values of the standard curve may vary according to the conditions of the actual assay performance (e.g. operator, pipetting technique, washing technique or temperature effects), the operator should establish a standard curve for each test. Typical standard curve and data is provided below for reference only.

| pg/mL | OD | Standard Curve |
|---|---|---|
| 100 | 0.232 | 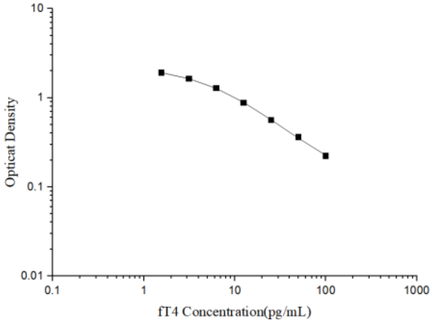 |
| 50 | 0.356 | |
| 25 | 0.563 | |
| 12.5 | 0.912 | |
| 6.25 | 1.259 | |
| 3.13 | 1.633 | |
| 1.56 | 1.905 | |
| 0 | 2.236 | |



## Performance

- **Precision**

Intra-assay Precision (Precision within an assay): 3 samples with low, mid range and high level fT4 were tested 20 times on one plate, respectively.

Inter-assay Precision (Precision between assays): 3 samples with low, mid range and high level fT4 were tested on 3 different plates, 20 replicates in each plate, respectively.

| | Intra-assay Precision | | | Inter-assay Precision | | |
|---|---|---|---|---|---|---|
| Sample | 1 | 2 | 3 | 1 | 2 | 3 |
| n | 20 | 20 | 20 | 20 | 20 | 20 |
| Mean(pg/mL) | 5.24 | 11.7 | 41.57 | 4.78 | 11.52 | 39.64 |
| Standard deviation | 0.22 | 0.59 | 2.18 | 0.41 | 0.95 | 3.27 |
| CV (%) | 4.2 | 5.05 | 5.25 | 8.6 | 8.23 | 8.24 |

## ■ Recovery

The recovery of fT4 spiked at three different levels in samples throughout the range of the assay was evaluated in various matrices.

| Sample Type | Range (%) | Average Recovery (%) |
|---|---|---|
| Serum (n=8) | 92-106 | 99 |
| EDTA plasma (n=8) | 90-101 | 96 |
| Cell culture media(n=8) | 96-107 | 102 |

## ■ Linearity

Samples were spiked with high concentrations of fT4 and diluted with Reference Standard & Sample Diluent to produce samples with values within the range of the assay.

| | | Serum (n=5) | EDTA plasma (n=5) | Cell culture media(n=5) |
|---|---|---|---|---|
| 1:2 | Range (%) | 87-101 | 99-112 | 91-104 |
| | Average (%) | 93 | 106 | 98 |
| 1:4 | Range (%) | 85-96 | 92-108 | 92-105 |
| | Average (%) | 90 | 98 | 100 |
| 1:8 | Range (%) | 88-104 | 85-98 | 94-109 |
| | Average (%) | 95 | 92 | 101 |
| 1:16 | Range (%) | 89-106 | 87-99 | 96-111 |
| | Average (%) | 97 | 94 | 103 |

## Declaration

1. Limited by current conditions and scientific technology, we can't conduct comprehensive identification and analysis on all the raw material provided. So there might be some qualitative and technical risks for users using the kit.
2. This assay is designed to eliminate interference by factors present in biological samples. Until all factors have been tested in the ELISA immunoassay, the possibility of interference cannot be excluded.
3. The final experimental results will be closely related to the validity of products, operational skills of the operators, the experimental environments and so on. We are only responsible for the kit itself, but not for the samples consumed during the assay. The users should calculate the possible amount of the samples used in the whole test. Please reserve sufficient samples in advance.
4. To get the best results, please only use the reagents supplied by the manufacturer and strictly comply with the instructions.
5. Incorrect results may occur because of incorrect operations during the reagents preparation and loading, as well as incorrect parameter settings of the Micro-plate reader. Please read the instructions carefully and adjust the instrument prior to the experiment.
6. Even the same operator might get different results in two separate experiments. In order to get reproducible results, the operation of every step in the assay should be controlled.
7. Every kit has strictly passed QC test. However, results from end users might be inconsistent with our data due to some variables such as transportation conditions, different lab equipment, and so on. Intra-assay variance among kits from different batches might arise from the above reasons too.
8. Kits from different manufacturers or other methods for testing the same analyte could bring out inconsistent results, since we haven't compared our products with those from other manufacturers.
9. The kit is designed for research use only, we will not be responsible for any issues if the kit is applied in clinical diagnosis or any other related procedures.
