# Supplementary material for: Identification of hub genes in congenital hypothyroidism and construction of the associated immune regulatory network
Source: Front Immunol. 2025 Oct 15;16:1608098. doi: 10.3389/fimmu.2025.1608098 (PMC12568518; doi:10.3389/fimmu.2025.1608098)
Supplement: Supplementary file 13 [file Table8.docx]

**STROBE-MR checklist of recommended items to address in reports of Mendelian randomization studies**^1^ ^2^

| **Item No.** | **Section** | **Checklist item** | **Page No.** | **Relevant text from manuscript** |
| --- | --- | --- | --- | --- |
| 1 | **TITLE and ABSTRACT** | Indicate Mendelian randomization (MR) as the study’s design in the title and/or the abstract if that is a main purpose of the study | 1 | Immunoinfiltration analysis was carried out to verify the immunological correlation. Inflammatory factors and immune cells were screened by batch Mendelian randomization. |
|  | **INTRODUCTION** |  |  |  |
| 2 | **Background** | Explain the scientific background and rationale for the reported study. What is the exposure? Is a potential causal relationship between exposure and outcome plausible? Justify why MR is a helpful method to address the study question | 2 | These findings strongly suggest that there is a close association between immunity and hypothyroidism (20) |
| 3 | **Objectives** | State specific objectives clearly, including pre-specified causal hypotheses (if any). State that MR is a method that, under specific assumptions, intends to estimate causal effects | 2 | First, bioinformatics, machine learning, and batch Mendelian randomization (MR) were used to screen and validate the hub genes and the related immune regulatory network. |
|  | **METHODS** |  |  |  |
| 4 | **Study design and data sources** | Present key elements of the study design early in the article. Consider including a table listing sources of data for all phases of the study. For each data source contributing to the analysis, describe the following: |  |  |
|  | a) | Setting: Describe the study design and the underlying population, if possible. Describe the setting, locations, and relevant dates, including periods of recruitment, exposure, follow-up, and data collection, when available. | N | Not performed |
|  | b) | Participants: Give the eligibility criteria, and the sources and methods of selection of participants. Report the sample size, and whether any power or sample size calculations were carried out prior to the main analysis | N | Not performed |
|  | c) | Describe measurement, quality control and selection of genetic variants | 4 | First, genome-wide significant SNPs (P < 5×10-8) were included. If there were no significant SNPs available as instrumental variables (IVs), SNPs with a P value less than the genome-wide significance level (P < 5×10-6) were considered as candidates. |
|  | d) | For each exposure, outcome, and other relevant variables, describe methods of assessment and diagnostic criteria for diseases | N | Not performed |
|  | e) | Provide details of ethics committee approval and participant informed consent, if relevant | 4 | All the data were derived from publicly available databases, and all the original studies obtained ethical approval. |
| 5 | **Assumptions** | Explicitly state the three core IV assumptions for the main analysis (relevance, independence and exclusion restriction) as well assumptions for any additional or sensitivity analysis | 4 | These SNPs were subsequently clustered according to linkage disequilibrium (window size = 10,000 kb, r2 < 0.001), and weak instrumental variables (F statistic < 10) were excluded. |
| 6 | **Statistical methods: main analysis** | Describe statistical methods and statistics used |  |  |
|  | a) | Describe how quantitative variables were handled in the analyses (i.e., scale, units, model) | N | Not performed |
|  | b) | Describe how genetic variants were handled in the analyses and, if applicable, how their weights were selected | 4 | First, genome-wide significant SNPs (P < 5×10-8) were included. If there were no significant SNPs available as instrumental variables (IVs), SNPs with a P value less than the genome-wide significance level (P < 5×10-6) were considered as candidates. These SNPs were subsequently clustered according to linkage disequilibrium (window size = 10,000 kb, r2 < 0.001), and weak instrumental variables (F statistic < 10) were excluded. |
|  | c) | Describe the MR estimator (e.g. two-stage least squares, Wald ratio) and related statistics. Detail the included covariates and, in case of two-sample MR, whether the same covariate set was used for adjustment in the two samples | 4 | Calculation of the F statistic strength: For each SNP included in the analysis, depending on the data situation, the following methods were used to calculate the R2 or F value. R2 = 2EAF(1-EAF)β2 or R2 = β2/(β2+SE2N), and F = R2(N-2)/(1-R2). Moreover, F ≥ 10 was required to evaluate the effectiveness of the instrument. |
|  | d) | Explain how missing data were addressed | N | Not performed |
|  | e) | If applicable, indicate how multiple testing was addressed | N | Not performed |
| 7 | **Assessment of assumptions** | Describe any methods or prior knowledge used to assess the assumptions or justify their validity | 2 | These findings strongly suggest that there is a close association between immunity and hypothyroidism (20). Therefore, determining whether there is an immune-related pathogenesis in CH is the focus of this study. |
| 8 | **Sensitivity analyses and additional analyses** | Describe any sensitivity analyses or additional analyses performed (e.g. comparison of effect estimates from different approaches, independent replication, bias analytic techniques, validation of instruments, simulations) | 4 | The P values for heterogeneity and horizontal pleiotropy were calculated. P > 0.5 indicated that there was no heterogeneity or pleiotropy in the samples. Any outliers were removed, and the MR-dependent estimates were re-evaluated. If the heterogeneity remained high after removing the outliers, a random effects model was used to assess the stability of the results. Finally, a leave-one-out analysis was conducted to verify the impact of each SNP on the overall causal relationship estimate. |
| 9 | **Software and pre-registration** |  |  |  |
|  | a) | Name statistical software and package(s), including version and settings used | 4 | we used the TwosampleMR package in R (version 4.4.1) to conduct MR analysis |
|  | b) | State whether the study protocol and details were pre-registered (as well as when and where) | N | Not performed |
|  | **RESULTS** |  |  |  |
| 10 | **Descriptive data** |  |  |  |
|  | a) | Report the numbers of individuals at each stage of included studies and reasons for exclusion. Consider use of a flow diagram | N | Not performed |
|  | b) | Report summary statistics for phenotypic exposure(s), outcome(s), and other relevant variables (e.g. means, SDs, proportions) | N | Not performed |
|  | c) | If the data sources include meta-analyses of previous studies, provide the assessments of heterogeneity across these studies | N | Not performed |
|  | d) | For two-sample MR:  i.  Provide justification of the similarity of the genetic variant-exposure associations between the exposure and outcome samples  ii.  Provide information on the number of individuals who overlap between the exposure and outcome studies | 2 | These findings strongly suggest that there is a close association between immunity and hypothyroidism (20). Therefore, determining whether there is an immune-related pathogenesis in CH is the focus of this study. |
| 11 | **Main results** |  |  |  |
|  | a) | Report the associations between genetic variant and exposure, and between genetic variant and outcome, preferably on an interpretable scale | 10 | IL-2 positively regulated (CD27 on IgD-CD38+ B cell) and (CD27 on switched memory B cell)/natural killer cell receptor 2B4 (CD244) and negatively regulated (CD33dim HLA DR+ CD11b - absolute count)/interleukin-1 (IL-18) and (CD28+ CD4-CD8-T-cell %T cell)/osteoprotegerin (OPG), thereby promoting the progression of CH. |
|  | b) | Report MR estimates of the relationship between exposure and outcome, and the measures of uncertainty from the MR analysis, on an interpretable scale, such as odds ratio or relative risk per SD difference | N | Not performed |
|  | c) | If relevant, consider translating estimates of relative risk into absolute risk for a meaningful time period | N | Not performed |
|  | d) | Consider plots to visualize results (e.g. forest plot, scatterplot of associations between genetic variants and outcome versus between genetic variants and exposure) | 10 | The forest plots, funnel plots, scatter plots, and leave-one-out forest plots for the four-step screening of 20 immune pathways are shown in Supplementary Figure S5 [A - S]. |
| 12 | **Assessment of assumptions** |  |  |  |
|  | a) | Report the assessment of the validity of the assumptions | N | Not performed |
|  | b) | Report any additional statistics (e.g., assessments of heterogeneity across genetic variants, such as *I^2^*, Q statistic or E-value) | N | Not performed |
| 13 | **Sensitivity analyses and additional analyses** |  |  |  |
|  | a) | Report any sensitivity analyses to assess the robustness of the main results to violations of the assumptions | 4 | The P values for heterogeneity and horizontal pleiotropy were calculated. P > 0.5 indicated that there was no heterogeneity or pleiotropy in the samples. |
|  | b) | Report results from other sensitivity analyses or additional analyses | 4 | Finally, a leave-one-out analysis was conducted to verify the impact of each SNP on the overall causal relationship estimate. |
|  | c) | Report any assessment of direction of causal relationship (e.g., bidirectional MR) | N | Not performed |
|  | d) | When relevant, report and compare with estimates from non-MR analyses | N | Not performed |
|  | e) | Consider additional plots to visualize results (e.g., leave-one-out analyses) | N | Not performed |
|  | **DISCUSSION** |  |  |  |
| 14 | **Key results** | Summarize key results with reference to study objectives | 10 | IL-2 positively regulated (CD27 on IgD-CD38+ B cell) and (CD27 on switched memory B cell)/natural killer cell receptor 2B4 (CD244) and negatively regulated (CD33dim HLA DR+ CD11b - absolute count)/interleukin-1 (IL-18) and (CD28+ CD4-CD8-T-cell %T cell)/osteoprotegerin (OPG), thereby promoting the progression of CH. |
| 15 | **Limitations** | Discuss limitations of the study, taking into account the validity of the IV assumptions, other sources of potential bias, and imprecision. Discuss both direction and magnitude of any potential bias and any efforts to address them | N | Not performed |
| 16 | **Interpretation** |  |  |  |
|  | a) | Meaning: Give a cautious overall interpretation of results in the context of their limitations and in comparison with other studies | N | Not performed |
|  | b) | Mechanism: Discuss underlying biological mechanisms that could drive a potential causal relationship between the investigated exposure and the outcome, and whether the gene-environment equivalence assumption is reasonable. Use causal language carefully, clarifying that IV estimates may provide causal effects only under certain assumptions | N | Not performed |
|  | c) | Clinical relevance: Discuss whether the results have clinical or public policy relevance, and to what extent they inform effect sizes of possible interventions | N | Not performed |
| 17 | **Generalizability** | Discuss the generalizability of the study results (a) to other populations, (b) across other exposure periods/timings, and (c) across other levels of exposure | N | Not performed |
|  | **OTHER INFORMATION** |  |  |  |
| 18 | **Funding** | Describe sources of funding and the role of funders in the present study and, if applicable, sources of funding for the databases and original study or studies on which the present study is based | 14 | This work was supported by the Shandong Maternal and Child Health Association, grant number SFYXH-2023W043. We also appreciate the support from the Weifang Youth Medical Talent Recommendation Program. |
| 19 | **Data and data sharing** | Provide the data used to perform all analyses or report where and how the data can be accessed, and reference these sources in the article. Provide the statistical code needed to reproduce the results in the article, or report whether the code is publicly accessible and if so, where | 4 | Data on CH were obtained from the IEU database (https://gwas.mrcieu.ac.uk/), specifically for hypothyroidism (congenital or acquired) (GWAS ID: finn-b-HYPOTHYROIDISM, containing 16,378,441 single nucleotide polymorphisms (SNPs)). A total of 728 types of immune cells were sourced from the EBI GWAS Catalog database (https://www.ebi.ac.uk/gwas/), corresponding to the GWAS ID (Supplementary Table S1-728). Ninety types of inflammatory factor data were obtained from the EBI GWAS Catalog, with corresponding IDs (Supplementary Tables S1-90). Data on IL-2 were obtained from the IEU database (GWAS ID: prot-c-3070_1_2, containing 501,428 SNPs). |
| 20 | **Conflicts of Interest** | All authors should declare all potential conflicts of interest | 14 | The authors declare that the research was conducted in the absence of any commercial or financial relationships. |

This checklist is copyrighted by the Equator Network under the Creative Commons Attribution 3.0 Unported (CC BY 3.0) license.

1. Skrivankova VW, Richmond RC, Woolf BAR, Yarmolinsky J, Davies NM, Swanson SA, et al. Strengthening the Reporting of Observational Studies in Epidemiology using Mendelian Randomization (STROBE-MR) Statement. JAMA. 2021;under review.

2. Skrivankova VW, Richmond RC, Woolf BAR, Davies NM, Swanson SA, VanderWeele TJ, et al. Strengthening the Reporting of Observational Studies in Epidemiology using Mendelian Randomisation (STROBE-MR): Explanation and Elaboration. BMJ. 2021;375:n2233.
